# Serum biomarkers identification by iTRAQ and verification by MRM: S100A8/S100A9 levels predict tumor-stroma involvement and prognosis in Glioblastoma

**DOI:** 10.1038/s41598-019-39067-8

**Published:** 2019-02-26

**Authors:** Anjali Arora, Vikas Patil, Paramita Kundu, Paturu Kondaiah, A. S. Hegde, A. Arivazhagan, Vani Santosh, Debnath Pal, Kumaravel Somasundaram

**Affiliations:** 1 Departments of Microbiology and Cell Biology, Indian Institute of Science, Bangalore, 560012 India; 2 Molecular Reproduction, Development and Genetics, Indian Institute of Science, Bangalore, 560012 India; 3 Computational and Data Sciences, Indian Institute of Science, Bangalore, 560012 India; 4 Sri Satya Sai Institute of Higher Medical Sciences, Bangalore, 560066 India; 5 Departments of Neurosurgery, National Institute of Mental Health and Neuro Sciences, Bangalore, 560029 India; 6 Departments of Neuropathology, National Institute of Mental Health and Neuro Sciences, Bangalore, 560029 India

## Abstract

Despite advances in biology and treatment modalities, the prognosis of glioblastoma (GBM) remains poor. Serum reflects disease macroenvironment and thus provides a less invasive means to diagnose and monitor a diseased condition. By employing 4-plex iTRAQ methodology, we identified 40 proteins with differential abundance in GBM sera. The high abundance of serum S100A8/S100A9 was verified by multiple reaction monitoring (MRM). ELISA and MRM-based quantitation showed a significant positive correlation. Further, an integrated investigation using stromal, tumor purity and cell type scores demonstrated an enrichment of myeloid cell lineage in the GBM tumor microenvironment. Transcript levels of S100A8/S100A9 were found to be independent poor prognostic indicators in GBM. Medium levels of pre-operative and three-month post-operative follow-up serum S100A8 levels predicted poor prognosis in GBM patients who lived beyond median survival. *In vitro* experiments showed that recombinant S100A8/S100A9 proteins promoted integrin signalling dependent glioma cell migration and invasion up to a threshold level of concentrations. Thus, we have discovered GBM serum marker by iTRAQ and verified by MRM. We also demonstrate interplay between tumor micro and macroenvironment and identified S100A8 as a potential marker with diagnostic and prognostic value in GBM.

## Introduction

Glioblastoma (GBM) is the most aggressive form of brain tumor in adults^1^. Despite various advancements in the understanding of this disease, the median survival is 14.6 months^2^. Early diagnosis and the appropriate prognosis are therefore important for effective treatment. WHO2016 classification included molecular features in addition to histopathology, which makes way for the future addition of other biomarkers for better management of GBM^3^.

Tumor and the host contribute to the proteins present in serum. Serum reflects tumor macroenvironment, therefore, serum proteins can be utilized as biomarkers to diagnose and monitor disease progression in a less invasive manner. Certain serum biomarkers for GBM like YKL40 and GFAP have been reported earlier but have shown limitations for clinical use^4–10^. Additionally, reciprocal interactions between tumor and its micro and macro-environment also play a decisive role in disease prognosis and have the potential for therapeutic intervention^11^.

To examine the role of GBM serum proteins to monitor and influence the disease progression, we performed an unbiased quantitative differential proteomics of GBM patient sera, using iTRAQ, which revealed several potential candidate biomarkers. We performed a thorough examination of association between GBM educated macro and microenvironment utilizing our iTRAQ data and transcripts of high abundant serum protein in tumor tissue from publically available datasets. We verified and validated two proteins S100A8 and S100A9 to be upregulated in GBM sera by MRM and ELISA. We further performed an in-depth investigation of their source of origin as well as diagnostic and prognostic capabilities of RNA and serum levels of these two proteins.

## Results

### Workflow of the biomarker study

**Figure 1:**
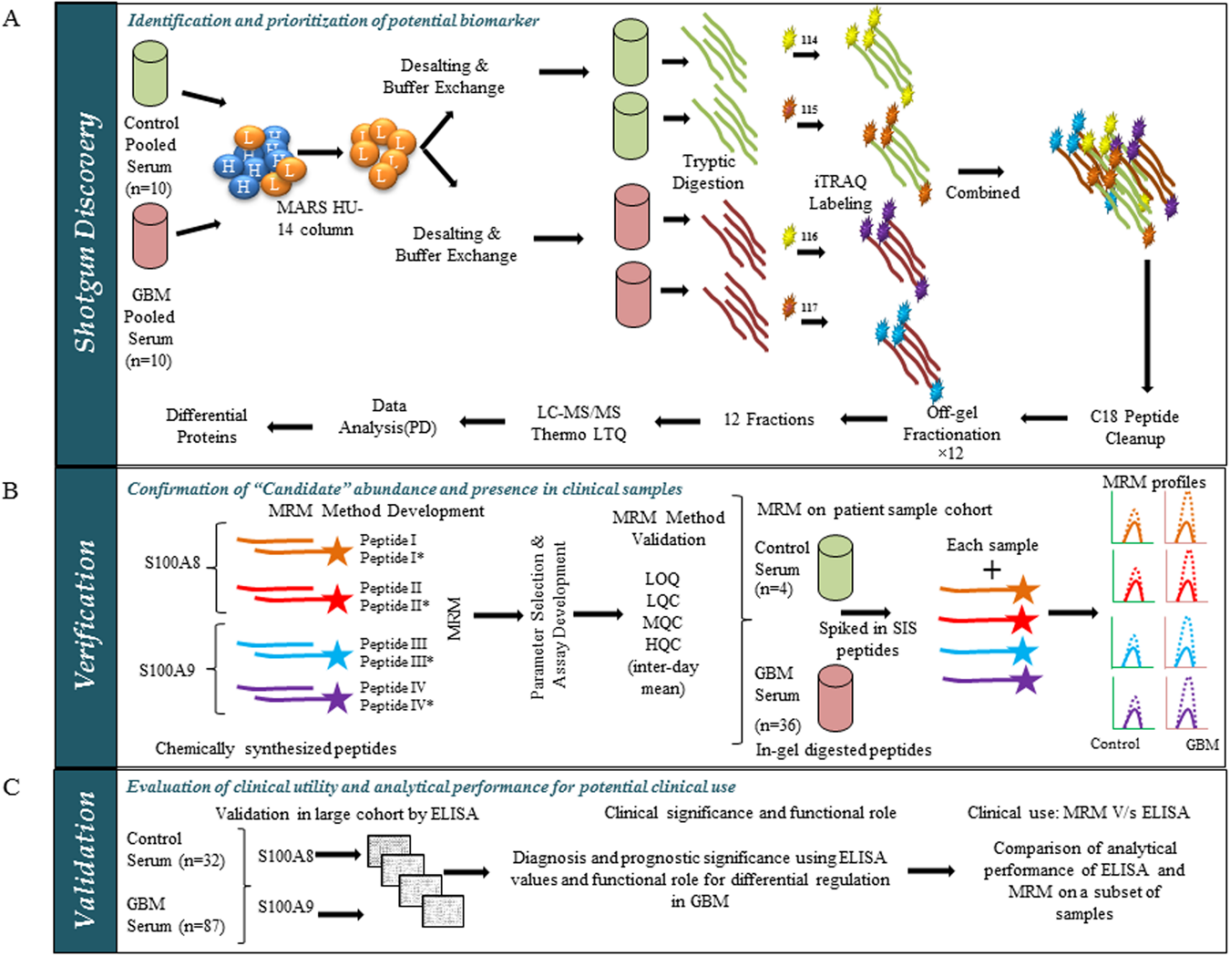
Schematic describing the serum biomarker study work flow. (**A**) Shotgun discovery: Control pooled sera (n = 10) and GBM pooled sera (n = 10) were subjected to depletion using HU-14 MARS column, to remove high abundant proteins (HAP, H in blue circle) and to obtain low abundant proteins (LAP, L in orange circle). LAP of pooled sera were tryptic digested and labeled using iTRAQ reagents 114 and 115 (yellow and orange) for duplicates of control serum and 116 and 117 (purple and sky blue) for duplicates of GBM serum. iTRAQ labeled control and GBM peptides were mixed together and subjected to PI based offgel fractionation. Fractions obtained (n = 12) were subjected to LC-MS/MS and the data obtained was analyzed by Proteome Discoverer (PD) to obtain differential abundant proteins in GBM sera. (**B**) Verification by MRM: MRM assay for S100A8 and S100A9, the two most abundant proteins, was developed using two peptides from each protein. Synthetic light peptides were used to optimize the parameters (shown in orange, red, sky blue and purple) and SIS peptides stable-isotope-labeled standard peptide (SIS peptides, shown in orange, red, sky blue and purple with star) were used as internal standards. Developed method was validated to check sensitivity and reproducibility by determining the standard measurements such as, limit of quantitation (LOQ), lower quality control (LQC), middle quality control (MQC), and higher quality control (HQC). This MRM assay was used to determine levels of S100A8 and S100A9 in GBM (n = 36) and control (n = 4) sera. Diagrammatic representation for MRM profile, showing equal SIS peptide in control and GBM (orange, red, sky blue and purple peaks, thick line), but high levels of endogenous peptide (orange, red, sky blue and purple peaks, dotted line) is shown. (**C**) Validation by ELISA: Serum from a large cohort was subjected to ELISA based measurement. Values obtained were used to perform statistical analysis for obtaining diagnostic and prognostic significance of S100A8 and S100A9. Functional role of higher abundance was investigated by *in vitro* assays. To evaluate the analytical performance of MRM assay, ELISA and MRM based quantitation of the same cohort was compared.

We present here a complete workflow of this biomarker study, which consists of three parts- Identification in the discovery phase, Verification of candidate biomarkers and Validation and analysis of clinical significance in a larger cohort (Fig. 1). This study design is in accordance with the proposed “NCI-CPTC Biomarker Development Pipeline”^12^. In the discovery phase, we performed shotgun proteomics of control and GBM pooled serum samples using untargeted 4-plex iTRAQ (isobaric Tags for Relative and Absolute Quantitation) mass spectrometry to identify candidate biomarkers with differential abundance (Fig. 1A). The two candidates, S100A8 and S100A9 were prioritized for verification based on their maximal abundance in GBM serum in our discovery phase. For the verification step, targeted proteomics was performed using MRM (Multiple Reactions Monitoring). An MRM based assay method specific for S100A8 and S100A9 was developed using synthetic light peptides. MRM assay for both the proteins was optimized for reproducibility by performing quality control measurements multiple times. Calibration curves were used for the absolute quantification with spiked in SIS (Stable-Isotope-labelled Standard) peptides for normalization. The developed MRM assay was used to measure the proteins in a cohort of control and GBM serum samples (Fig. 1B). We then carried out ELISA based validation in a larger cohort with clinical information. Common samples between ELISA and MRM were used to compare the output of two methods. Serum protein levels and tumor transcript levels of S100A8 and S100A9 were used for detailed analysis to probe the role of these two proteins in GBM diagnosis and prognosis. The significance of high levels of S100A8 and S100A9 in glioma biology was established by performing *in vitro* functional assays (Fig. 1C).

### Serum biomarker identification for GBM by isobaric Tags for Relative and Absolute Quantification (iTRAQ)

**Figure 2:**
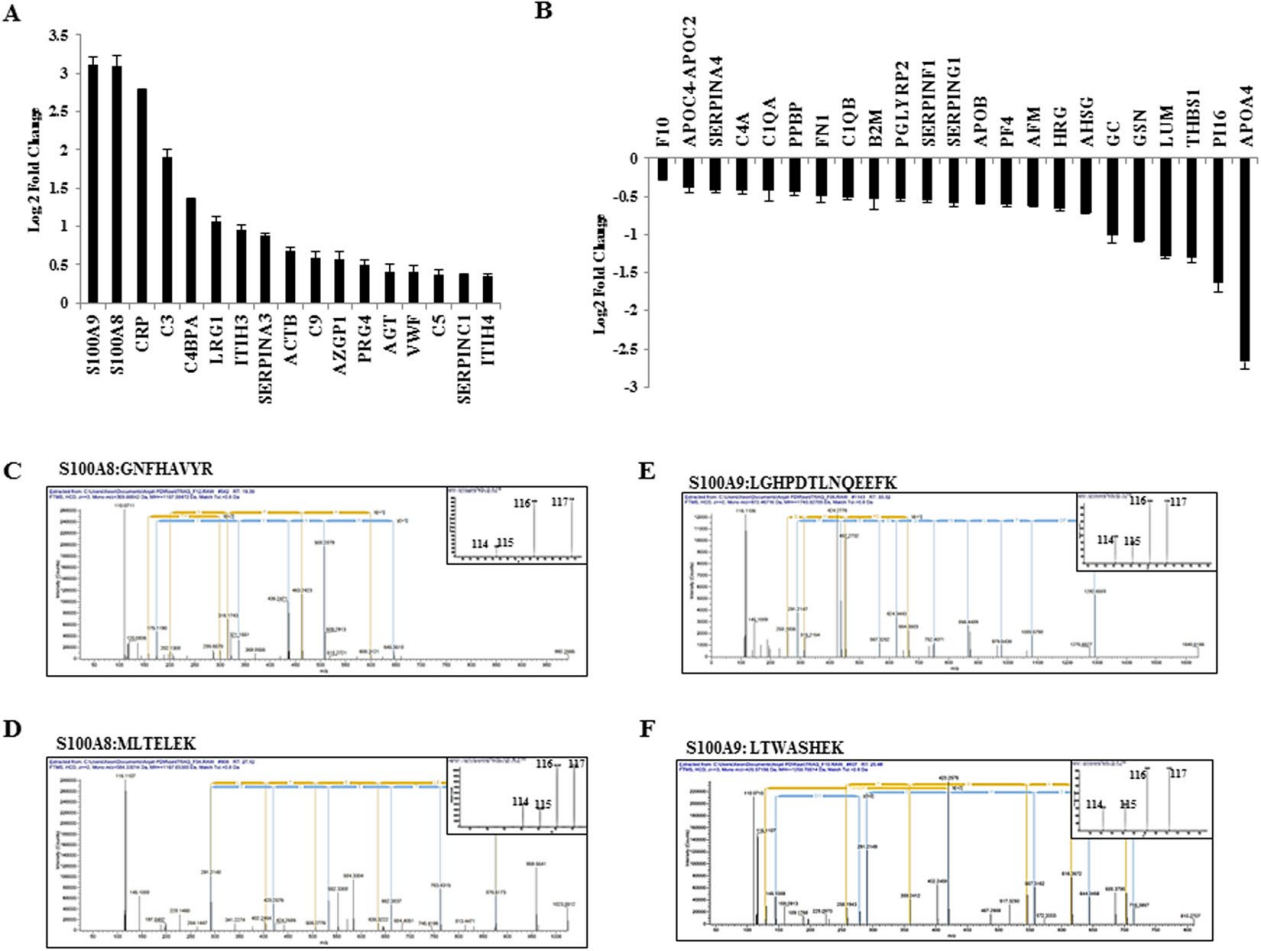
Proteins with differential abundance in GBM serum identified by iTRAQ. (**A**,**B**) Log_2_ transformed ratios for proteins with at least two unique peptides having high abundance and low abundance, respectively, in GBM sera as compared to control sera are shown. (**C**,**D**) Representative tandem mass spectrometry (MS/MS) spectra for peptide sequence GNFHAVY and MLTELEK derived from S100A8. iTRAQ label 114 and 115 were used for control pooled serum sample and 116 and 117 were used for GBM pooled serum sample. (**E**,**F**) Representative tandem mass spectrometry (MS/MS) spectra for peptide sequence LGHPDTLNQEEFK and LTWASHEK derived from S100A9. iTRAQ label 114 and 115 were used for control pooled serum sample and 116 and 117 were used for GBM pooled serum sample.

We performed shotgun proteomics of serum samples from GBM sera and control sera using 4-plex iTRAQ to identify candidate biomarkers. iTRAQ labeled peptides from both samples were mixed, depleted, fractionated and subjected to LC-MS/MS (Supplementary Figs 1 and 2, details are given in the supplementary information). A total of 102 proteins with at least two unique peptides respectively at FDR < 1% were detected. Further analysis identified 40 proteins with two unique peptides to have differential abundance between control and GBM sera (Supplementary Table 1A,B, Fig. 2A,B). Two most abundant proteins S100A8 and S100A9 were chosen for verification and testing their clinical relevance and functional importance. MS/MS spectra for two peptides of S100A8 and S100A9 are shown (Fig. 2C–F).

### Verification of S100A8 and S100A9 as high abundant proteins in GBM sera by Multiple Reaction Monitoring (MRM) and Validation by ELISA

For verification of iTRAQ data, we developed an MRM assay to measure S100A8 and S100A9 (details are given in the supplementary information). Two peptides for each of the proteins were selected and three transitions per peptide were optimized (Table 1, Supplementary Fig. 3A–D). The assay was found to be reproducible as the percentage coefficient of variation for standard measurements was found to be minimal (Supplementary Table 2). A cohort of GBM (n = 36) and control (n = 4) serum samples were subjected to developed MRM assay to measure the endogenous peptides using their corresponding calibration curves. The linearity of developed method was achieved with r² > 0.99 for all four peptides (Supplementary Fig. 4A,B). While the MRM profile of control samples showed very low or undetectable levels for all four peptides, their presence in GBM samples was readily detected to varying levels. However, all samples showed significant and equivalent detection of spiked in stable-isotope-labeled standard peptides (SIS peptides), which were used for ratio calculations towards normalization (Supplementary Fig. 4C,D).

**Table 1:** MRM parameters for light and SIS peptides.

S. No.	Name	Sequence	Polarity	Precursor ion (m/z)	Product ion (m/z)	Collision Energy	S-lens Voltage	Retention time (min)
**MRM parameters for light and SIS peptides of S100A8**								
1	Peptide I	GNFHAVYR	_+_ve	482.24	508.16	20	110	7.7
2		GNFHAVYR	_+_ve	482.24	645.46	20	110	7.7
3		GNFHAVYR	_+_ve	482.24	792.61	20	110	7.7
4	Peptide I*	GNFHAVYR*	_+_ve	487.28	*518.26*	20	95	7.7
5	Peptide II	MLTELEK	_+_ve	432.23	518.21	15	90	8.5
6		MLTELEK	_+_ve	432.23	619.35	15	90	8.5
7		MLTELEK	_+_ve	432.23	732.41	15	90	8.5
8	Peptide II*	MLTELEK*	_+_ve	436.4	*627.36*	15	90	8.5
**MRM parameters for light and SIS peptides of S100A9**								
9	Peptide III	LGHPDTLNQGEFK	_+_ve	728.36	936.31	25	148	8.2
10		LGHPDTLNQGEFK	_+_ve	728.36	1148.7	25	148	8.2
11		LGHPDTLNQGEFK	_+_ve	728.36	1310.68	25	148	8.2
12	Peptide III*	LGHPDTLNQGEFK*	_+_ve	732.5	*1156.5*	25	125	8.2
13	Peptide IV	LTWASHEK	_+_ve	486.25	571.52	20	111	7.9
14		LTWASHEK	_+_ve	486.25	757.38	20	111	7.9
15		LTWASHEK	_+_ve	486.25	858.01	20	111	7.9
16	Peptide IV*	LTWASHEK*	_+_ve	490.27	*765.26*	20	91	7.9

*Indicates the stable-isotope-labelled standard (SIS) peptides; Bold in the peptide amino acid sequence refers to the labelled amino acid residue; Italic figures indicates transition ion used for quantitation.

**Figure 3:**
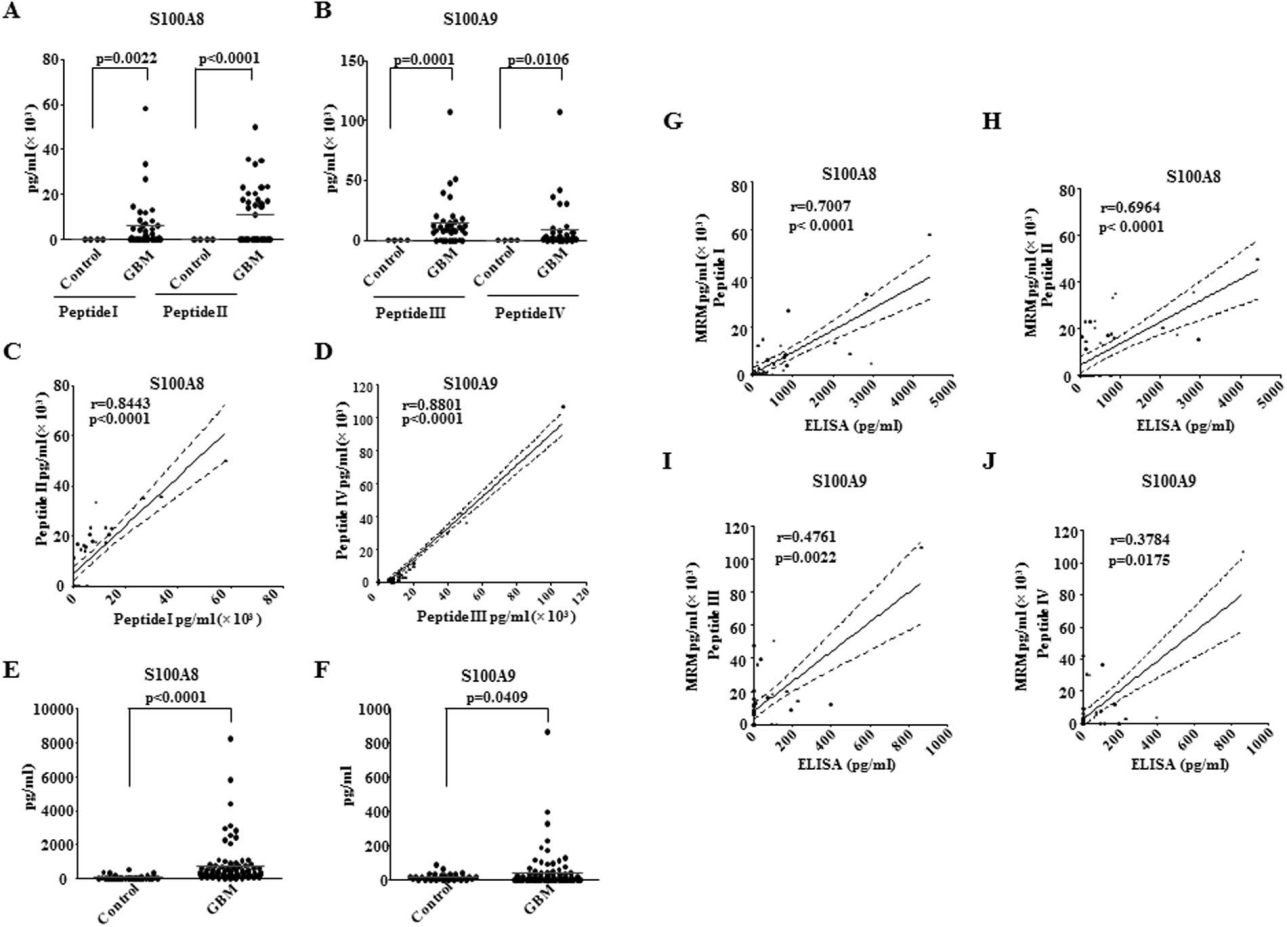
Verification of S100A8 and S100A9 by MRM and Validation by ELISA. (**A**,**B**) The concentrations obtained by MRM for two peptides of S100A8 and S100A9, in GBM (n = 36) as compared to control sera (n = 4) are plotted. p values calculated by unpaired t test with Welch’s correction are indicated. p value less than 0.05 is considered significant with *, **, *** representing p value less than 0.05, 0.01 and 0.001 respectively. (**C**,**D**) The correlation plots between two peptides of S100A8 and two peptides of S100A9, is shown. Correlation coefficient and p value, calculated by Spearman’s correlation are indicated, dotted lines represent 95% confidence interval. (**E**,**F**) Serum levels S100A8 and S100A9 respectively were measured by ELISA on a larger cohort for validation (Control n = 32, GBM n = 87). p values calculated by unpaired t test with Welch’s correction are indicated. (**G**,**H**) Concentrations of S100A8 in GBM serum, obtained from MRM (Peptide I and Peptide II) were correlated with ELISA based values in the same cohort. Correlation coefficient and p value, calculated by Spearman’s correlation are indicated, dotted lines represent 95% confidence interval. (**I**,**J**) Concentrations of S100A9 in GBM serum, obtained from MRM (Peptide III and Peptide IV) were correlated with ELISA based values in the same cohort. Correlation coefficient and p value, calculated by Spearman’s correlation are indicated, dotted lines represent 95% confidence interval.

There was a significant high abundance of both peptides for S100A8 and S100A9 (Mean values for peptide I, II, III and IV in GBM: 6504.9,11311.7,14812.1,9071.5 pg/ml respectively) in GBM sera as compared to non-detectable values in control sera as measured by MRM thus verified the results of discovery phase (Fig. 3A,B). The values obtained by MRM for two peptides for S100A8 (Peptide I and Peptide II) and S100A9 (Peptide III and Peptide IV) also showed significant positive correlation (Fig. 3C,D). Next, ELISA based validation was carried out in a larger cohort (Fig. 1C). The values obtained by ELISA validated the high abundance of S100A8 and S100A9 in GBM sera (Fig. 3E,F). The serum levels of S100A8 and S100A9 obtained by ELISA and MRM showed significant positive correlation thus confirming the robustness of the MRM assay (Fig. 3G–J). Thus, MRM assay for the measurement of S100A8 and S100A9 was developed with comparable analytical performance with ELISA.

### GBM sera proteome analysis reveals tumor-driven micro and macroenvironment

**Figure 4:**
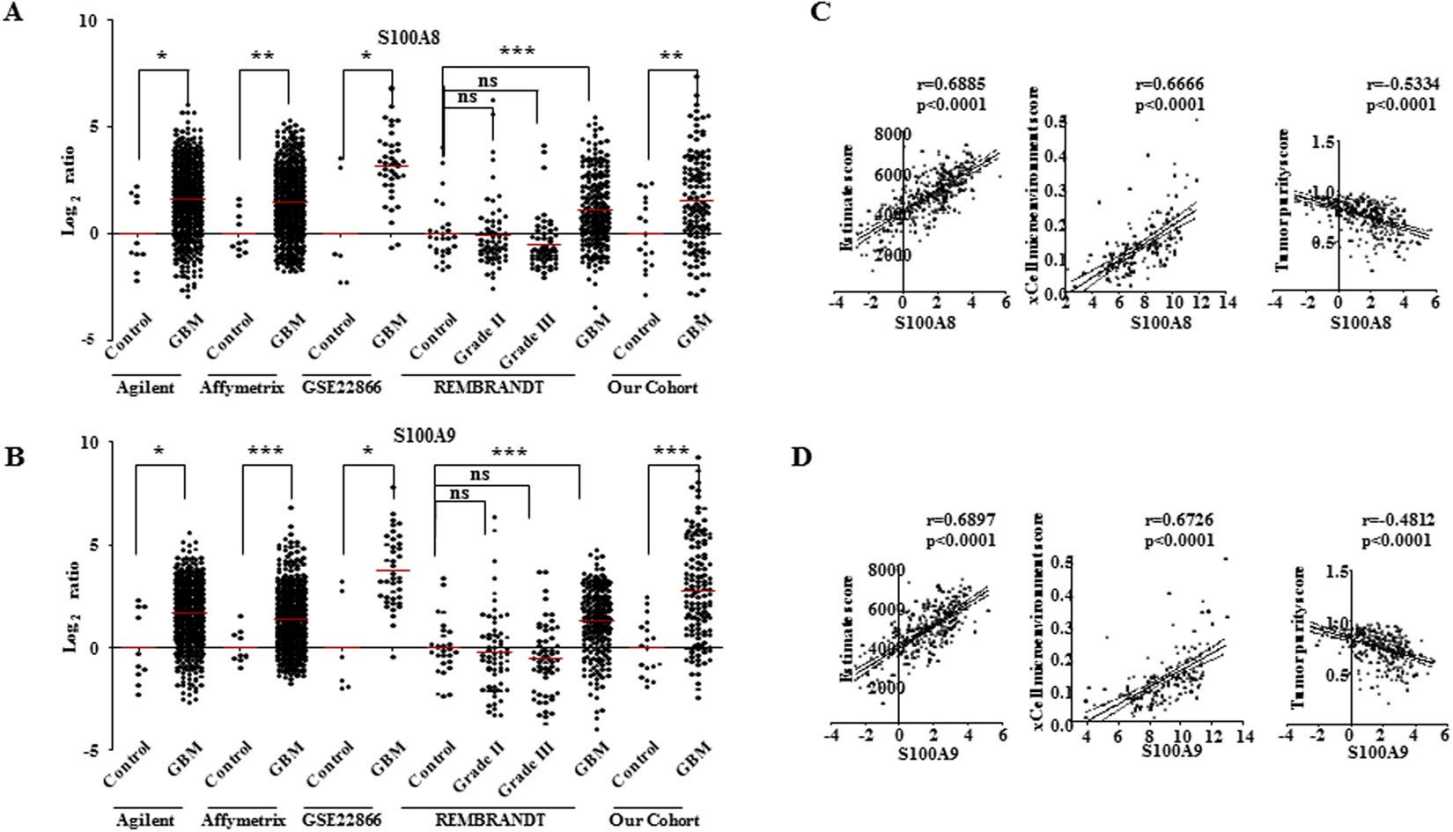
Potential involvement of microenvironment in the high levels of S100A8 and S100A9 transcripts in GBM tumor. (**A**,**B**) Transcript levels derived from publically available microarray data sets and our cohort (RT-qPCR) for S100A8 and S100A9 respectively, in control (TCGA Agilent n = 10, TCGA Affymetrix n = 10, GSE22866 n = 6, Rembrandt n = 28) and in GBM samples (TCGA Agilent n = 572, TCGA Affymetrix n = 528, GSE22866 n = 40, REMBRANDT Grade II n = 65, Grade III n = 58, GBM n = 227) are shown as scatter plot. Unpaired t-test with Welch’s correction was performed between control brain and GBM samples, p values are indicated with *, **, *** representing p value less than 0.05, 0.01 and 0.001 respectively. (**C**) Correlation of transcript level of S100A8 in TCGA dataset with ESTIMATE score, xCell microenvironment score and ABSOLUTE-based tumor purity score is shown. Correlation coefficient and p values are indicated, calculated by Spearman’s correlation, dotted lines represent 95% confidence interval. (**D**) Correlation of transcript level of S100A9 in TCGA dataset with ESTIMATE score, xCell microenvironment score and ABSOLUTE-based tumor purity score is shown. Correlation coefficient and p values are indicated, calculated by Spearman’s correlation, dotted lines represent 95% confidence interval.

It has been shown that secreted factors from the tumor cells modulate the conditions at distant sites, for example bone marrow and spleen, which results in the pathological differentiation of myeloid cells such as macrophages, dendritic cells, and granulocytes thus creating an altered tumor-driven macroenvironment^13^. The altered macroenvironment also results in the accumulation of immunosuppressive myeloid cells in the tumor microenvironment which supports neovascularization and metastasis^13^. Accordingly, we next dissected the contribution of GBM tumor and its microenvironment in determining the composition of GBM macroenvironment as represented by the altered GBM sera proteome. To test this, firstly, we checked tumor transcript levels of GBM serum high abundant proteins in TCGA transcriptome dataset. We found a good fraction of them (8/17 is 47%) to be upregulated at RNA level in tumor tissue compared to control brain samples (Supplementary Table 3). Notably, the transcript levels of S100A8 and S100A9 were found to be significantly up-regulated in GBM tumors as compared to lower grade glioma and control brain tissue in multiple datasets and our cohort (Fig. 4A,B). Secondly, we correlated the transcript levels of high abundant sera proteins to (1) ESTIMATE (Estimation of STromal and Immune cells in MAlignant Tumors using Expression data) score^14^, (2) xCell microenvironment score^15^, both of which measures the extent of stromal component thus are together called as “stromal scores” in this study and (3) ABSOLUTE-based tumor purity score^16^, which measures the extent of tumor cell component. The transcript levels of a significant number of serum proteins with high abundance (7/17 is 41%) in GBM sera were found to significantly correlate with stromal scores and with tumor purity score in different datasets (Supplementary Table 4). It is interesting to note that the transcript levels of all of these seven genes had positive significant correlation with stromal scores and tumor purity score respectively thus strengthening the fact that tumor-microenvironment has a significant impact on the composition of GBM sera proteome. This analysis also reveals that the transcripts of these proteins in heterogeneous tumor tissue are associated with the extent of stromal infiltration. In particular, we noted that S100A8 and S100A9 transcript levels in GBM have a significant, positive, high correlation with stromal scores and a significant, negative, correlation with tumor purity score (Fig. 4C,D). These results along with high transcript levels of these two proteins seen in tumor suggest a microenvironment origin to these two transcripts seen in the tumor. The high correlation also indicated that S100A8 and S100A9 transcripts have potential to serve as surrogate markers for levels of stromal infiltration in tumor tissue.

These results warranted further investigation of cells types in the tumor microenvironment which are contributing to transcripts of high abundant serum proteins in tumor tissue. We used xCell method which differentiates between six subgroups consisting of 64 cell types, using transcriptome data on the basis of cell-type specific gene signatures^15^. We performed correlation between cell type-specific signatures and transcript levels of the high abundant serum proteins that showed significant correlation with stromal and tumor purity scores. As expected, we found that the tumor transcript levels of 7 proteins (that showed a positive correlation with stromal scores) showed a significant positive correlation with most myeloid group-specific cell-type signatures and a significant negative correlation with most lymphoid group-specific cell-type signatures (Supplementary Fig. 5A, Supplementary Table 5). Furthermore, a strong significant positive correlation of S100A8 and S100A9 transcript was also found with myeloid cell surface antigen CD33 transcript (Supplementary Fig. 5B,C).

We also found S100A8 and S100A9 transcript level to be significantly high in mesenchymal GBM subtype, which is known for macrophages/microglia infiltration (Supplementary Fig. 6A,B)^17^. The high levels of S100A8 and S100A9 transcript seen in GBM was restricted to IDH1 wild-type GBM subgroup, which is a more aggressive type of GBM^18,19^ (Supplementary Fig. 6C,D). We also found stromal scores to be maximal in mesenchymal sub-group and IDH1 wild-type GBM group indicating the association of these two GBM subgroups with maximal stromal involvement (Supplementary Fig. 6E–H). In support of this, tumor purity score was found to be the minimum in mesenchymal and IDH1 wild-type GBM subgroups (Supplementary Fig. 6I,J). These results demonstrate that high levels of S100A8 and S100A9 seen in GBM sera is more likely contributed by tumor cells orchestrated tumor micro and macroenvironment.

### Diagnostic and Prognostic significance of S100A8 and S100A9 in GBM

**Figure 5:**
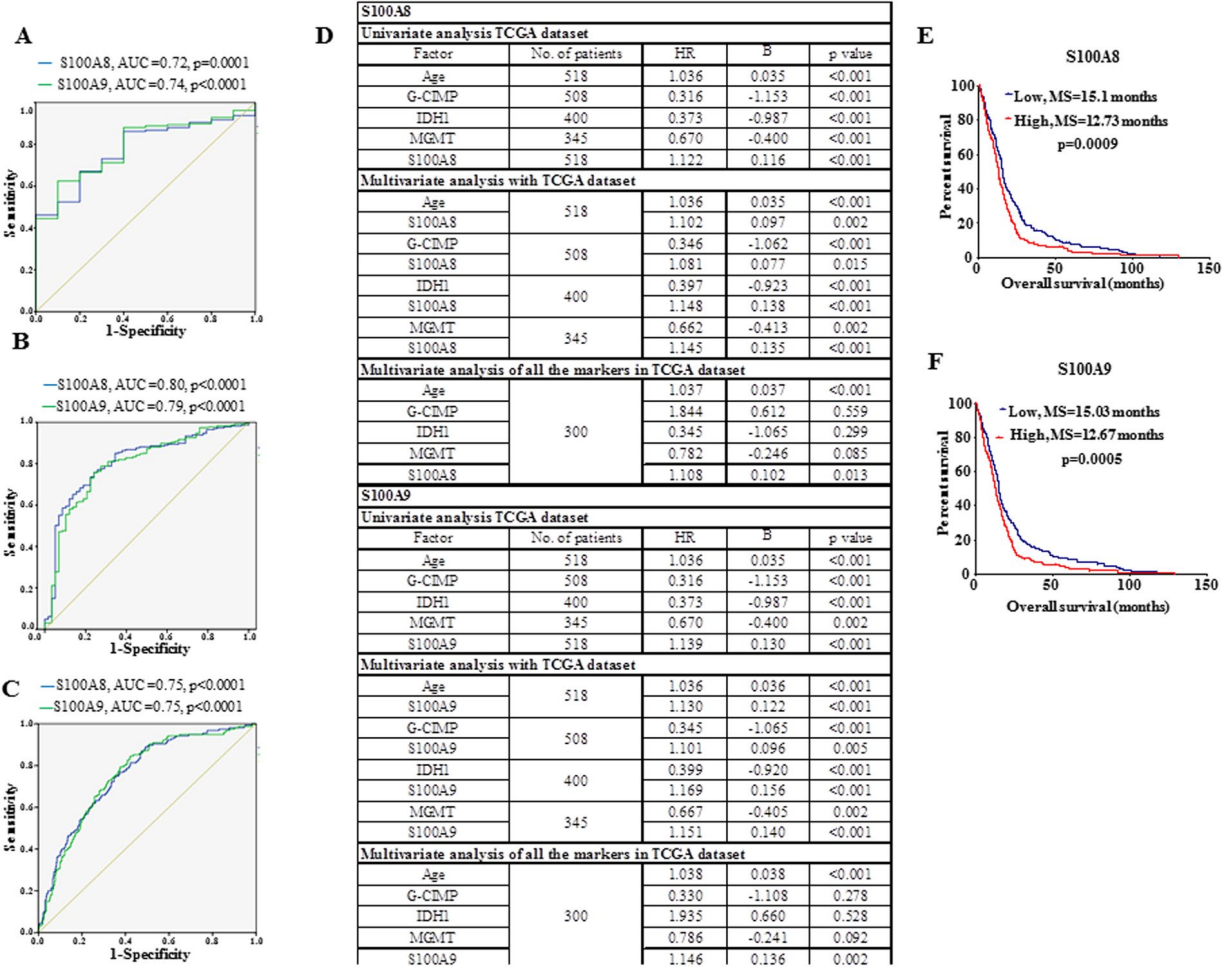
S100A8 and S100A9 transcripts are diagnostic and poor prognostic markers for GBM. (**A**) ROC curve depicting transcript levels of S100A8 and S100A9 discriminate between control and GBM, AUC and p value is indicated. (**B**) ROC curve depicting transcript levels of S100A8 and S100A9 discriminate between grade III and GBM, AUC and p value is indicated. (**C**) ROC curve depicting transcript levels of S100A8 and S100A9 discriminate mesenchymal subtype from other GBM subtypes, AUC and p value is indicated. (**D**) Univariate and Multivariate cox proportional hazard regression analysis was performed using TCGA Affymetrix data for transcript levels of S100A8 and S100A9 along with age, G-CIMP status, IDH1 mutation status, MGMT promoter methylation status. (**E**,**F**) Kaplan Meier survival analysis using TCGA Affymetrix cohort (n = 518) for transcript levels of S100A8 and S100A9. Log-rank (Mantel–Cox) test was applied and the p value is indicated.

We evaluated the diagnostic and prognostic value of S100A8 and S100A9 transcript levels using publically available GBM datasets. As the transcript levels of S100A8 and S100A9 were found to be upregulated significantly in GBM as compared to grade III (Fig. 4A,B), we evaluated the performance of these two transcript to discriminate GBM from control brain and found area-under-curve (AUC) value of 0.72 and 0.74 respectively (p = 0.0001, p < 0.0001, Fig. 5A). Further, S100A8 and S100A9 were also able to significantly differentiate between grade III and GBM with an AUC value of 0.80 and 0.79 respectively (p < 0.0001, p < 0.0001, Fig. 5B). In addition to this, S100A8 and S100A9 transcripts were able to discriminate mesenchymal group from other GBM subtypes with an AUC of 0.75 and 0.75 respectively (p < 0.0001, p < 0.0001, Fig. 5C). The Univariate Cox regression analysis revealed that S100A8 and S100A9 transcript levels predicted poor prognosis in GBM (Fig. 5D). Multivariate Cox regression analysis with age, G-CIMP, IDH1 mutation, MGMT promoter methylation revealed that the S100A8 and S100A9 transcript levels to be independent predictors of survival in GBM (Fig. 5D). Kaplan-Meier survival analysis showed that patients having higher S100A8 or S100A9 transcript levels have significantly lesser median survival (Fig. 5E,F). Thus, we found that tumor S100A8 and S100A9 transcript levels as independent poor prognosticators in GBM.

**Figure 6:**
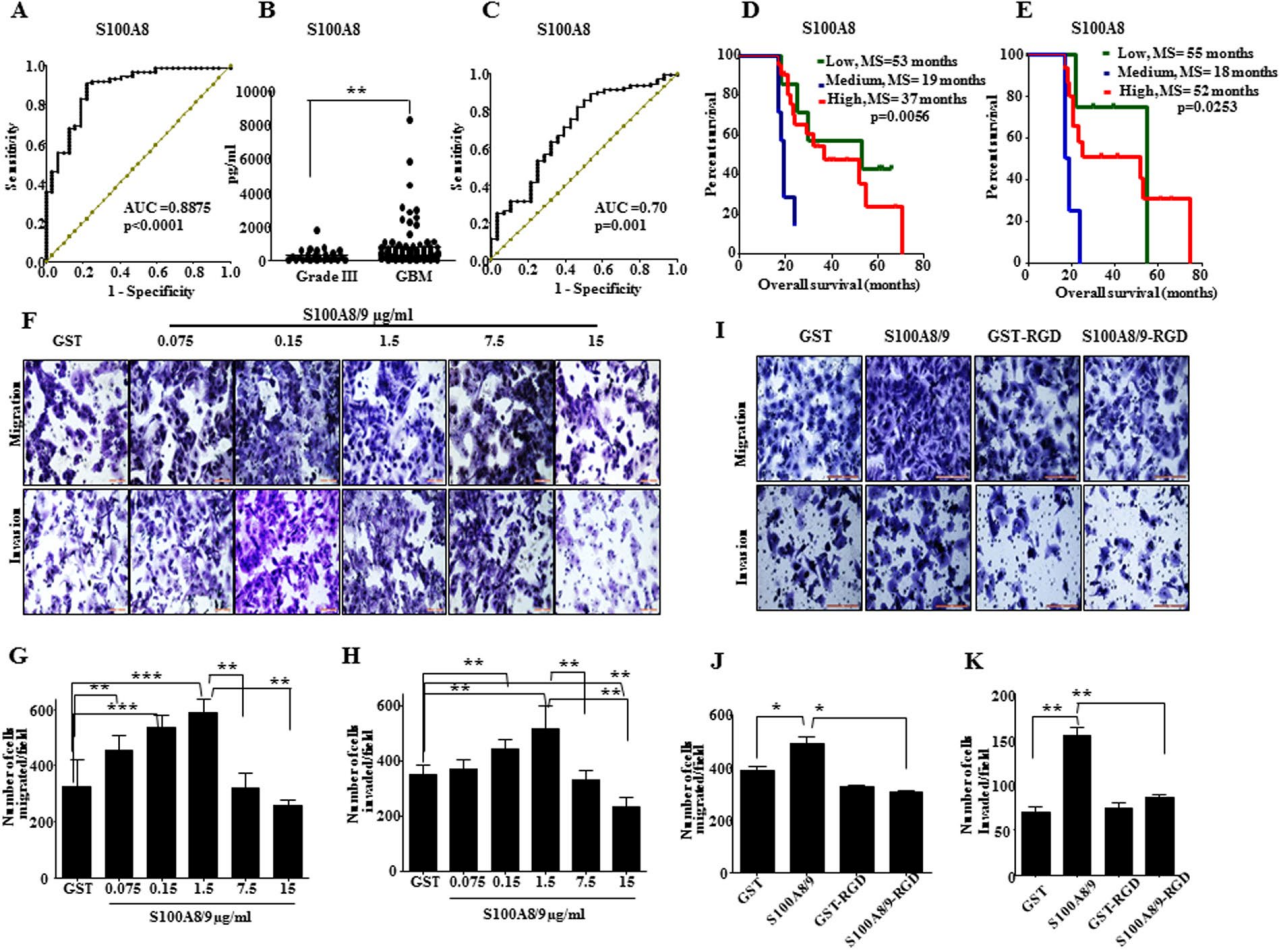
Serum levels of S100A8 serve as discriminatory and prognostic marker for GBM: (**A**) ROC curve depicting serum levels of S100A8 discriminate between healthy control and GBM is plotted, AUC and p value is indicated. (**B**) Scatter plot showing high abundance of serum S100A8 in GBM as compared to grade III as measured by ELISA. (**C**) ROC curve depicting serum levels of S100A8 discriminate between grade III and GBM, AUC and p value is indicated. (**D**) Kaplan Meier survival analysis using pre-operative serum levels of GBM patients surviving more than median survival (n = 35). Log-rank (Mantel–Cox) test was applied and the p value is indicated. (**E**) Kaplan Meier survival analysis using three months post-operative serum levels of GBM patients surviving more than median survival (n = 23). Log-rank (Mantel–Cox) test was applied and the p value is indicated. (**F**) Concentration dependent role on migratory and invasive property of exogenously added rS100A8 and rS100A9 (recombinant proteins) was measured using trans-well assay. Representative images of U373 cells fixed and stained after migration and invasion respectively are shown. (**G**,**H**) Quantitation for migration and invasion capability of U373 in presence of increasing concentration of exogenous rS100A8 and rS100A9. (**I**) Effect of integrin signaling inhibiting peptide, RGD (20 μM/ml), on migratory and invasive property of U373 cells in presence of exogenously added rS100A8 and rS100A9 (0.5 μg/ml) was measured using trans-well assay. Representative images of U373 cells fixed and stained after migration and invasion respectively are shown. (**J**,**K**) Quantitation for migration and invasion capability of U373 cells in presence of exogenous rS100A8 and rS100A9 (0.5 μg/ml) and RGD peptide (20 μM/ml).

Next, we investigated the diagnostic and prognostic capability of serum S100A8 and S100A9 levels (Supplementary Table 6). Receiver Operating Curve (ROC) analysis established that serum S100A8 levels produced high area-under-curve (AUC) value of 0.888 (p < 0.0001), thus demonstrating the discriminatory power of S100A8 (Fig. 6A). However, the serum levels of S100A9 failed to serve as a discriminatory marker (Supplementary Fig. 7A). We further performed ROC analysis using grade III and GBM serum levels of S100A8 and found it to be a discriminatory marker for the same with AUC value of 0.70 (p = 0.001) (Fig. 6B,C). Kaplan Meier survival analysis revealed that pre-operative serum levels of S100A8 and S100A9 failed to predict survival (Supplementary Fig. 7B,C). However, when only patients who survived more than median survival were considered, three groups with differing serum S100A8 levels showed a significant difference in survival (Fig. 6D; p = 0.0056, S100A9 makes only two groups with non-significant prognosis, Supplementary Fig. 7D). Interestingly, the group with the medium level of serum S100A8 showed worst prognosis (median survival 19 months) compared to groups with low and high levels of serum S100A8 (median survival 53 and 37 months respectively). In the same cohort, 3 months post-operative follow-up serum level of S100A8 also showed similar results (Fig. 6E, n = 23; p = 0.02). These results suggest that medium range of serum S100A8 predicts poor prognosis. Thus, we found that GBM serum S100A8 levels predict poor prognosis in long term survivors.

### Extracellular S100A8 and S100A9 induce glioma cell migration and invasion by inducing Integrin signalling

To investigate the functional role of S100A8 and S100A9 in GBM microenvironment, we studied the effect of recombinant S100A8 and S100A9 on various proprieties of glioma cells. While the exogenous addition of S100A8 and S100A9 did not show any change in proliferation as measured by colony formation (Supplementary Fig. 8A,B), there was an increase in migration of glioma cells (Supplementary Fig. 8C–F). The concentration-dependent prognosis by S100A8 encouraged us to inspect the effect of concentration of S100A8 and S100A9 proteins on the observed phenotype. We found the addition of increasing amounts of S100A8 and S100A9 proteins increased migration and invasion in a concentration-dependent manner up to a threshold level, after which they failed to enhance the migration and invasion in U373 cells (Fig. 6F–H). Similarly, S100A8 and S100A9 proteins induced migration and invasion of U138 glioma cells at a medium, but not at a higher concentration (Supplementary Fig. 8G–I). To further analysis the down-stream signaling activation, we probed for various pathways known to promote migration and invasion in glioma cells. We found S100A8 and S100A9 failed to induce glioma cell migration and invasion in cells treated with integrin inhibitory peptides (RGD) (Fig. 6I–K). These results indicate that S100A8 and S100A9 promote glioma cells migration and invasion through integrin signaling pathway.

## Discussion

Blood is the specialized body fluid that delivers the necessary molecules to almost every tissue in the human body and carries away the metabolic by-products along with it. In this process, it also collects some of the proteins from various parts of the body representing the current condition of metabolism and could be used as biomarkers of diseases. These proteins change in concentration or state in association with the specific biological process or disease. The relative abundance of these proteins, present originally in circulation or arising from different parts of the body as a result of the diseased condition, can be studied in a less-invasive manner by testing and studying human serum. Serum biomarkers can help in early detection, diagnosis, risk prediction, the prognosis of cancer, monitoring of therapeutic response, and studying the overall tumor macroenvironment. The challenge lies in finding biomarkers from serum, and its establishment in a larger cohort. We used iTRAQ for an unbiased discovery of candidate biomarkers in GBM sera. We identified 40 proteins with two unique peptides to have differential abundance between control and GBM sera. The top high abundant proteins in GBM sera identified included S100A9 and CRP, which have been shown previously to be higher in GBM patients^20^. CRP has also been shown to be of diagnostic importance^21^. AHSG was found to be a low abundant protein in GBM sera in this study has been reported to be a poor prognosticator in GBM^10^. At the core of a study, which deals with the serum biomarker discovery of a disease condition in a high throughput manner, exist, two important issues: the verification and validation of the discovery phase data and ease of developing an assay that can bring the biomarker to the clinics. MRM has been appreciated in recent times as a method of biomarker verification with high sensitivity and reproducibility^12^. MRM is increasingly gaining an advantage over ELISA method because of its multiplexing capability. Where ELISA has to be done separately for different proteins needed to be measured in a given sample, MRM can easily measure up to 20 to 30 proteins simultaneously in one sample^22^. With the initial cost of mass spectrometer establishment in a clinical setting, a developed assay for a biomarker in the pre-clinical study can be translated to patients in a very cost-effective manner. MRM also overcomes the limitations of antibody unavailability and time required for assay development in ELISA. We verified our iTRAQ data by developing MRM assay for the two most abundant proteins S100A8 and S100A9 in GBM sera. We provide the complete assay parameters required for the measurement of these two proteins in serum. The MRM output was found positively correlating to the measured values by ELISA. These finding indicate towards the potential of usage of MRM based assays in clinics for various diagnosis and prognostic tests.

S100A8 and S100A9 are members of S100 multigene subfamily of cytoplasmic EF-hand Ca+ binding proteins. These two proteins are expressed at different levels in different tissue types with their higher expression in myeloid cells but not in lymphocytes^23^. While these proteins are known to perform intracellular functions such as calcium sensing, they are also released in extracellular milieu by a less elucidated mechanism as both proteins do not contain trans-membrane signaling region^24^. Both are known to have differential expression in various malignancies and inflammatory conditions^25–28^. In prostate cancer, benign prostate hyperplasia, and colorectal cancer, serum S100A8 and S100A9 are shown to have diagnostic potential^29,30^. In invasive ductal carcinoma, the expression of S100A8 and S100A9 are suggested to be poor prognostic^31^. In GBM, although protein levels of S100A8 and S100A9 were found to be elevated in serum and tissue by various studies^20,32–34^, their therapeutic and functional role has not been established. Hence, in this study, we attempted to correlate the elevated levels of S100A8 and S100A9 transcripts in the GBM tissue and the proteins in serum with the patient prognosis. We found upregulated transcript levels of S100A8 and S100A9 in GBM tissue. It has been reported that S100A8 and S100A9 are frequently co-expressed and their expression is co-regulated^35^. Accordingly, we found a significant positive correlation between S100A8 and S100A9 transcripts and serum proteins (Supplementary Fig. 6K). As mentioned above, it is known that these two proteins form heterodimer. We performed Protein-Protein Interaction analysis of differential serum proteins and found while many of them interact with other proteins extensively, S100A8 and S100A9 formed a secluded module with limited number of interactions (Supplementary Fig. 9, Supplementary Table 7).

The differential GBM serum proteome is a depiction of tumor macroenvironment. We questioned whether the differential proteins in GBM sera are contributed by tumor cells or its microenvironment. The analysis of TCGA transcript levels of high abundant proteins found in our study revealed that a good fraction of them, including S100A8 and S100A9, showed higher transcripts in GBM and significant positive correlation with stromal scores and negative correlation with tumor purity score. The correlation indeed provided an insight into the real source of transcript upregulation. This finding is supported by a recent study where it has been demonstrated that the cell origin of S100A8 and S100A9 in GBM tissue as polymorphonuclear-myeloid derived suppressor cells (PMN-MDSCs)^32^, which are known to contribute to the suppressive immune environment^36^. The shedding of exosomes from murine MDSCs has also been suggested as source of S100A8 and S100A9^37^. Further analysis using xCell method revealed the maximum correlation of transcripts of high abundant serum proteins, including S100A8 and S100A9, with myeloid lineage and a negative correlation with cells signature of lymphoid lineage, suggesting the presence of higher levels of S100A8 and S100A9 transcript in tumor tissue can serve as indicator of a biased myeloid differentiation of tumor micro and macroenvironment. We found the transcript levels of S100A8 and S100A9 to be significantly higher in mesenchymal GBM subtype, which is reported to be enriched with macrophages/microglia infiltration^17^, and in IDH1 wild-type subgroup, known to be more aggressive and have a poor prognosis^18,19^. We also found enrichment of stromal scores in mesenchymal and IDH1 wild-type GBM subgroup, suggesting increased S100A8 and S100A9 transcript in these subgroups could be due to increased microenvironment component. It has been reported that GBM has enhanced immune phenotype as compared to lower grades and high tumor purity is a good prognostic marker in IDH1 wild-type GBM^38,39^. This overall suggests the possible association of microenvironment with the aggressiveness of IDH1 wild-type and mesenchymal subtypes. To further utilize the fact that the transcripts of S100A8 and S100A9 are upregulated in GBM and specifically mesenchymal subtype of GBM we established their diagnostic capability to discriminate GBM from control brain and grade III gliomas and also mesenchymal subgroup from other subgroups within GBM. Higher expression levels of S100A8 and S100A9 in these subgroups also suggest they could serve as associated markers for better survival prediction. Indeed, we showed S100A8 and S100A9 transcript levels, independently can serve as a poor prognosticator in GBM. These results also indicate that the publically available data is derived from a heterogeneous population of cell types from tumor tissue and hence should be used with precaution^40^. As we establish, higher expression of S100A8 and S100A9 is indicative of the presence of pro-tumorigenic microenvironment and also predict prognosis, their expression levels in the post-operative tumor have the possibility to serve as a biomarker for predicting survival^41^.

S100A8 and S100A9 are known to have a dual role in tumor progression^42^. S100A8 and S100A9 have been shown to be pro-tumorigenic by inducing accumulation of MDSCs at the tumor site^43,44^ and enhancing angiogenesis^45^, on the other hand, have proven to be immune activating by enhancing NK cells activity^46^. They are also proved to be pro-apoptotic at higher concentration^47,48^ but pro-migratory at lower concentrations for various tumor types^49,50^. It has been discussed elsewhere, that there exists a balance between the pro-tumorigenic and anti-tumorigenic properties of S100A8 and S100A9 and which side the balance tilts is decided by levels and location of S100A8 and S100A9 expression^46^. S100B, another member of S100 family, also has been shown to have similar functions^51^. In our study, serum levels of both S100A8 and S100A9 were found to be higher, but only S100A8 served as a discriminatory marker. Serum levels of S100A8 and S100A9 failed to serve as a prognostic indicator in our complete cohort. However, when patients who survive more than median survival were considered, S100A8 serum levels could predict poor prognosis at medium levels with pre-operative as well as three months postoperative serum. Additionally, we found at medium concentration, but not at higher concentration, of S100A8 and S100A9 promoted migration and invasion, which was found to be dependent on integrin signaling. This suggests systemic levels of pre-operative and postoperative serum levels of S100A8 could be decisive in aggressiveness of GBM.

We represent a clear evidence of S100A8 and S100A9 is a poor prognosticator at higher transcript levels and their association with the microenvironment. For serum protein levels, the diagnostic and prognostic significance were found for S100A8. Although the results shown here are promising for consideration of S100A8 as a serum biomarker, the limitation is a high abundance of this protein in other neurological disorders with similar symptoms as GBM. Therefore, examination of serum levels of S100A8 in other neurological and inflammatory disorders would be required before it could be considered for GBM discrimination. S100A8 and S100A9 are known to form a functional heterodimer. We did not find serum levels of S100A9 showing diagnostic or prognostic significance. Our ELISA quantification had overall lower levels of S100A9 (100 × 103 pg/ml) than S100A8 (1000 × 103 pg/ml) (Fig. 3E,F). This could also be a reason for S100A9 did not give significance in our analysis. There is also a possibility of their independent functions, which have not been explored in this study.

In summary, in this study we provide evidence for increased expression of S100A8 and S100A9 in GBM patients both in the tissue and proteins in the serum. We also show association of S100A8 and S100A9 with enhanced stromal/immune infiltration and myeloid differentiation. Furthermore, the elevated transcripts of S100A8 and S100A9 predicted poorer prognosis and on the other hand, only S100A8 serum levels correlated with survival of patients.

## Material and Methods

### Tumor and serum samples used

Tumor and blood samples of GBM patients were collected from National Institute of Mental Health and Neurosciences (NIMHANS) and Sri Satya Sai Institute of Higher Medical Sciences (SSSIHMS) in Bangalore, India. The samples were obtained after an informed, written consent from all the patients, prior to the initiation of the study, in accordance with the NIMHANS and SSSIHMS institutional ethics committee (IEC) guidelines and approval. Tissues samples were used for RNA isolation (n = 130) and blood samples were used for obtaining serum (n = 125). Histological confirmation was centrally reviewed as GBM by the neuropathologist as per WHO 2007 classification scheme 58. A subset of patients was prospectively recruited. As controls for RNA, non-tumorous brain tissue during intractable epilepsy surgery were used (n = 18). Control blood samples were collected from healthy individuals at IISc Bangalore (n = 42). The sample size for all experiments was random unless specified. Other details provided in the supplementary information.

### Biomarker study workflow

Our study consisted of three parts: discovery phase using iTRAQ, verification by MRM and validation by ELISA. Details of all the steps followed are provided in the supplementary information. In short, in the discovery phase, GBM and control pooled sera were depleted using HU-14 MARS column and were labeled using 4-plex iTRAQ reagents. Offgel fraction was performed and mixed samples were subjected to LC-MS/MS on a Thermo LTQ. Data analysis was performed using proteome discoverer. Peptides with 1% FDR correction were used for identification and quantification. The two candidates, S100A8 and S100A9, were verified using multiple reactions monitoring (MRM). An MRM based assay specific for S100A8 and S100A9 was developed. MRM assays for both the proteins were optimized for reproducibility by performing standard measurements multiple times. Calibration curves were used for the absolute quantification with spiked in SIS peptides for normalization. The developed MRM assay was used to measure the proteins in a cohort of control (n = 4) and GBM (n = 36) serum samples.

ELISA based validation was performed for a larger cohort with clinical information (Control n = 32, GBM; n = 87). Non-linear regression analysis was performed for standard curve fitting. Inter- and intra-batch variations were calculated for comparison of values from different batches. Levels obtained from ELISA and MRM were compared. Clinical data of our cohort with serum levels and TCGA cohorts with transcript levels of S100A8 and S100A9 was used to probe the role of these two proteins at transcript level and serum protein level in GBM diagnosis and prognosis. The significance of high levels of S100A8 and S100A9 in glioma biology was established by performing functional assays. Section wise details are provided in the supplementary information.

### Survival analysis and statistics

Survival and ROC analysis were performed using SPSS software version 19 (IBM Cor., New York, USA). Confidence intervals and significance are provided. Significance difference between two groups of unequal population was performed by a nonparametric t-test with Welch’s correction. Multiple comparison analysis was performed by one-way ANOVA. Unpaired t-test was performed for *in-vitro* experiments with equal sample size. Spearman correlation method was used. Significance values below 0.05 were considered significant. Everywhere, two-sided statistics was performed.

## Supplementary information

Supplementary Information

Supplementary Table 1

Supplementary Table 2

Supplementary Table 3

Supplementary Table 4

Supplementary Table 5

Supplementary Table 6

Supplementary Table 7

## Data Availability

The mass spectrometry proteomics data have been deposited to the ProteomeXchange Consortium via the PRIDE^52^ partner repository with the dataset identifier PXD012207.
