# Supplementary material for: Serum biomarkers identification by iTRAQ and verification by MRM: S100A8/S100A9 levels predict tumor-stroma involvement and prognosis in Glioblastoma
Source: Sci Rep. 2019 Feb 26;9:2749. doi: 10.1038/s41598-019-39067-8 (PMC6391445; doi:10.1038/s41598-019-39067-8)
Supplement: Supplementary file 1 — Supplementary Information [file 41598_2019_39067_MOESM1_ESM.pdf]

Supplementary information:

Serum biomarkers identification by iTRAQ and verification by MRM: S100A8/S100A9 levels predict tumor-stroma involvement and prognosis in Glioblastoma

Anjali [1]Arora, Vikas [1]Patil, Paramita [2]Kundu, Paturu [2]Kondaiah, AS [4]Hegde, A [5]Arivazhagan, Vani [6]Santosh, Debnath [3]Pal and Kumaravel [1*]Somasundaram
[1]Departments of Microbiology and Cell Biology, [2]Molecular Reproduction, Development and Genetics, [3]Computational and Data Sciences, Indian Institute of Science, Bangalore 560012; [4]Sri Satya Sai Institute of Higher Medical Sciences, Bangalore 560066; Departments of [5]Neurosurgery and [6]Neuropathology, National Institute of Mental Health and Neuro Sciences, Bangalore 560029.

* Corresponding author
Tel: +91-80-23607171
Fax: +91-80-23602697
Email: skumar1@iisc.ac.in, ksomasundaram1@gmail.com

Supplementary materials and methods 2

Supplementary Figure legends 15

Supplementary Figures 19

Material and methods

Cell lines and reagents

Human glioma cell lines U373, U138, T98G, U87 were grown in Dulbecco's Modified Eagle Medium (DMEM) supplemented with 10% Fetal Bovine Serum (FBS) and antibiotics- penicillin and streptomycin. Reagents used in this study are as follows: TEAB (Triethyl ammonium bicarbonate) buffer (Sigma), iTRAQ Reagent Four-Plex Kit (Applied Biosystems), Trypsin Gold (modified sequencing grade trypsin; Promega, Madison, WI, USA), mass spectrometry grade water (Fluka), Off-gel fractionation reagents (Agilent), unlabelled synthetic light peptides (Cellmano Biotech Limited), Stable-isotope-labelled standard peptide (SIS-peptides, JPT Peptide Technologies), S100A8 and S100A9 ELISA kits (R & D systems; DY4570-05 and DY5578 respectively). Recombinant S100A8 and S100A9 were bacterially purified (details given in separate section).

Tumor/Serum sample collection and patient cohort characteristics:

Our prospective study included a total number of 154 patients who underwent surgical treatment at National Institute of Mental Health and Neurosciences and Sri SatyaSai Institute of Higher Medical Sciences, Bangalore, India between July 2006 and September 2009.All these patients were histologically proven to be GBM. The approval for this study id given by the ethics committee of NIMHANS (NIMHANS/IEC/No. RPA/060/05 dated 29.10.2005) and SSSIHMS (IEC No RPA/001/2005 dated 20.10.05) and written consent of patients was obtained. In this cohort all the patients were adults (age>18 yrs of age) with newly diagnosed GBM. All patients were subjected to total or near total excision of the tumor. The exclusion of the patients was done in case of previous surgery or recurrence. Patients were included in the study only if they have post-operative Karnofsky's Performance Score (KPS) ≥70. The treatment strategy followed for all the patients was same, which included radiotherapy (total dose of 59.4 Gy, given in 33 fractions) with concomitant temozolomide (100 mg/day for 45 days), followed by five cycles of temozolomide at a dose of 150 mg/sq. m body surface area. Follow up serum samples were collected at regular intervals with documentation of their clinical status. Overall survival was noted as the duration between surgery and death of the patient due to the disease. Of these 154

patients, serum samples were available for a subset of patients, which were considered for further analysis in our study.

For serum preparation, blood samples were allowed to clot at 4 °C overnight followed by collection of upper phase as serum after centrifugation for 5 min at 1000 rpm to separate serum at 4 °C and were stored at -80°C until used.

Sample preparation, iTRAQ labelling and Off-gel fractionation

A pool of 10 serum samples from GBM patient (GBM pooled sera) and 10 serum samples from healthy individual (control pooled sera), all age and gender matched (average control age=49.5 years, average GBM age =53.1 years, p=0.11, see below), was examined for differential expression of proteins. In order to remove high abundant proteins (HAP) from sera, which could mask the detection of low abundant proteins (LAP), both, control pooled sera and GBM pooled sera, separately, were subjected to depletion by affinity chromatography using MARS HU-14 column (4.6×100 mm; Agilent Technologies, Santa Clara, CA, USA). The terminology LAP refers to serum depleted of only fourteen high abundant proteins against which antibodies are present in the column. Flow-through fraction was collected as LAP **(Supplementary Figure 1A).** Buffer exchange and desalting was performed for LAP using 500mM TEAB buffer (Sigma) and 3kDa cut off centricon (Millipore) as per the manufacturer's instruction. Silver staining was performed to visualize the depletion of HAP **(Supplementary Figure 1B).**

To perform iTRAQ labelling, equal amount of LAP protein extract from GBM and control samples was separately subjected to tryptic digestion as per manufactures protocol in duplicates (Applied Biosystems iTRAQ Reagent Four-Plex Kit protocol). Briefly, 50 µg of LAP from control pooled sera and GBM pooled sera were reduced alkylated and tryptic digested for 18 hours at 37°C. iTRAQ reagents 114 and 115 were used to label two control duplicate samples and iTRAQ reagents 116 and 117 were used to label two GBM duplicate samples. iTRAQ labelled peptides from all four samples were mixed together, vacuum dried and were subjected to isoelectric point (PI) based fractionation using 3100 OFFGEL Fractionator kit (Agilent Technologies, Böblingen, Germany) with a setup of 24-well and an IPG strip of 24 cm, 3-10 linear pH range using manufacturer's protocol. Before electrofocusing, iTRAQ mixed sample was desalted using C18 spin columns (89873, Pierce). 24 fractions obtained after off-gel

fractionation were pooled into 12 fractions. The pooling of fractions was done such that all fractions should be having approximately equivalent representation of number of peptides. To aid to this decision of fraction pooling, theoretical digestion of 1929 proteins reported in human plasma proteome[1] was performed and the theoretical PI of the obtained peptides was calculated using EXPASY tool (http://web.expasy.org/peptide_mass/). Thus, all the peptides were binned into 24 fractions where they are expected to be present theoretically as per their PI (**Supplementary Figure 2A**). After doing this exercise, fractions with lesser peptides were pooled together, and fractions with higher number of peptides were left as single fraction (**Supplementary Figure 2B**). This exercise helped us to bring down the number of samples to be analysed from 24 to 12 with logic of equivalent peptide representation in all fractions. These 12 fractions were subjected to LC-MS/MS and the data was obtained. The number of proteins and peptides obtained in each fraction, in contrast to theoretically expected distribution, showed equivalent representation in all fractions, with a mean peptide value and a mean protein value with very low standard deviation (**Supplementary Figure 2C-E**).

**Clinical information of the pooled serum samples used for iTRAQ**			
S. No.	Sample	Gender	Age
1	GBM	M	46
2	GBM	M	60
3	GBM	M	51
4	GBM	M	54
5	GBM	M	55
6	GBM	F	55
7	GBM	F	60
8	GBM	F	50
9	GBM	F	55
10	GBM	F	45
11	Normal	M	48
12	Normal	M	57.5
13	Normal	M	50
14	Normal	M	41
15	Normal	M	51
16	Normal	F	50
17	Normal	F	54
18	Normal	F	48

19	Normal	F	45
20	Normal	F	51

LC-MS/MS

After off-gel fractionation and pooling, samples were desalted using C18 spin columns, separately for each fraction (89873, Pierce), vacuum dried and subjected separately for LC-MS/MS. LTQ-Orbitrap XL (Thermo) coupled with Agilent's 1200 Series nano-flow LC system was used. LTQ-Orbitrap was operated in a data-dependent mode, that is, one MS1 FTMS scan precursor ions followed by CID (collision induced dissociation) and HCD (higher energy collisional dissociation) MS2 scans of the five most abundant doubly or triply charged ions in each FTMS scan. The dried samples were reconstituted in 100 µL of the 5% acetonitrile and 0.1% formic acid and 1 µL of the same is injected on column. Digested peptides were subjected to 110 minute RPLC-MS/MS analysis (gradient given below).

	**NanoPump (300nl/min)**		**Capillary pump (20µl/min)**	
**S. No**	**%B**	**Time**	**%B**	**Time**
1	11	0	1	0
2	11	5	1	5.5
3	25	45	50	8
4	53	77	50	80
5	100	86	100	89
6	100	90	100	93
7	11	94	1	95
8	11	110	1	110

Mobile phase A: 100 % water with 0.1% Formic Acid and Mobile phase B: 80% acetonitrile + 20% water with 0.1%formic acid was used.

Protein identification and quantification

For protein identification and quantitation, individual and merged analysis with raw files of 12 fractions was performed using Proteome Discoverer version 1.4.0.288 (Thermo Scientific). Database search was carried out against the NCBI human RefSeq database using the SEQUEST search algorithm. The search parameters used were as follows: 1) precursor mass tolerance=12ppm, 2) fragment mass tolerance=0.8 Da, 3) fixed modification of iTRAQ4plex / +144.102 Da at peptide N-Terminus (any N-Terminus), 4) dynamic modification of N-Terminal Acetyl / +42.011 Da (Any N-Terminus), iTRAQ4plex / +144.102 Da (K), Carbamidomethyl / +57.021 Da (C), Oxidation / +15.995 Da (M), Phospho / +79.966 Da (S, T), Carbamyl / +43.006 Da (K), Deamidated / +0.984 Da (N), 5) maximum missed cleavage sites=3, 6) enzyme=trypsin. Unique peptides only and 1% false discovery rate (FDR) criteria were used for peptide filtering. Percolator algorithm (Proteome Discoverer version 1.4) was used to assigns a statistically meaningful q-value to each PSM. Medium-confidence PSMs (default) were considered. Best-scoring PSMs and the matches that have very similar scores were selected by using the Delta Cn peptide filter, wherein a Delta Cn better than 0.15 (default) was used. Peptide length filter of 7 aminoacid was applied. Relative quantification of proteins was performed based on the relative intensities of reporter ions generated during MS/MS fragmentation of peptides. “Normalize on protein median” was used under the settings of Quantification Method Editor/Experimental bias provided in Proteome Discoverer version 1.4**.** Final ratios (116/114 and 117/115) were obtained and average of the technical duplicates was used to plot the graph. A cut off of 20% was applied for coefficient of variation between the technical duplicates. For ratio calculation only unique peptides were used.

Multiple reaction monitoring

Two peptides for each protein were selected as target sequences for MRM **(Supplementary Figure 3A, 3B**). The selection of the proteotypic peptide was done on the basis of identification of the peptide in our iTRAQ data and availability of the peptides as being used for SRM before reported in SRMatlas (www.srmatlas.org)[2]. Synthetic peptides for the selected four sequences were obtained in both unlabelled (light peptides) and stable-isotope-labelled standard peptide

(SIS peptides). The identity of the synthesized peptides was confirmed by performing MS and MS/MS **(Supplementary Figure 3A, 3B).** All stocks and dilutions were prepared using 0.5% acetonitrile containing 0.1% formic acid as diluent. 10mg/ml stocks of the standards and 5mg/ml stocks of the internal standards were prepared by reconstitution of the lyophilized powder in suitable volume of diluent. Aliquots were made and stored at -80°C. From these, peptide I and peptide IV, were diluted to 10 µg/ml while peptide II and peptide III were diluted to 100 µg/ml each. Likewise, peptide I* and peptide IV* were diluted to 10 µg/ml each while peptide II* and peptide III* were diluted to 100 µg/ml each. Dilutions were decided on the basis of lower detection limit of these peptides in test runs.

Next, a mixture of all the light synthetic peptides in diluent containing, 2.5 µg/ml each of peptide I and peptide IV and, 25µg/ml each of peptide II and peptide III, respectively, was prepared. This was the highest standard and was diluted serially, to construct an 8-point calibration curve in which the SIS peptide concentration was held constant and the light peptide concentration was varied by appropriately diluting the light standard peptides. The SIS peptide mix of peptide I*, peptide IV*, peptide II*, peptide III* was prepared in the diluent to achieve concentrations of 1.25, 1.25, 12.5 and 12.5 µg/ml respectively.10 µl each of standard mix was diluted to 200 µl of 75% acetonitrile, dried and reconstituted in 40 µl of diluent. 10 µl of internal standard mix was spiked, mixed well and 10 µl was injected into LC-MS. Final amount of the highest standard on column was as follows: peptide I and peptide IV (5ng each) and peptide II and peptide III (50 ng each). Amount of internal standard on column was as follows: peptide I* and peptide IV* (2.5 ng each) and peptide II* and peptide III* (25ng each). The concentration of internal standard peptide is accurately known, thus the concentration of the protein to be measured is determined in the unknown sample by peak area ratio. MRM assay was developed by optimizing parameters like collision energy and retention time for three transitions per peptides, which were selected on the basis of product ion intensities obtained after performing MRM of light peptides. For each peptide, three transitions were measured, out of which one was used as the quantifier and the other two were used as qualifier to confirm the retention time and identity. For the assessment of reproducibility and sensitivity of the developed assay, limit of quantitation (LOQ), lower quality control (LQC), middle quality control (MQC) and higher quality control (HQC) were determined and nine repeats of the calibration curve over three days

was performed. The co-efficient of variation and accuracy was calculated by obtaining inter-day mean.

After method development, a cohort of control (n=4) and GBM (n=36) serum samples was subjected to MRM. After protein estimation, 3mg of each sample was loaded on the 18 % gel SDS-PAGE gel. After commassie staining, area between 10 to 16 kDa was subjected to in-gel tryptic digestion. Briefly, after obtaining the gel piece between 10-16 kDa for all the samples, destaining of coommassie was performed. Then the gel piece was reduced, alkylated and tryptic digested for 18 hours. Tryptic digestion was stopped by 1% formic acid. The peptides were extracted from the gel piece by 60% acetonitrile followed by 90% acetonitrile. The extracted peptides were vacuum dried and stored in -80°C until used. Before performing MRM, samples were reconstituted into 40 µl of diluent, and 10 µl of heavy standard peptide mix of peptide I*, peptide IV*, peptide II*, peptide III* were spiked in.

An Agilent 1290 Infinity UHPLC system with an analytical column (Agilent SB - C18, 1.8u, 100mmx2.1mm) was used for the separation of the peptides at a flow rate 0.2 ml /min. A gradient of 0-3mins:0.5%B, 3-12mins:0.5-50%B, 12-12.1mins:50-85%B, 12.1-15mins:85%B, 15-15.1mins:85-0.5%B, 15.1-20mins:0.5%B, where mobile phase A was 10mM Ammonium Acetate in Water (0.1%FA) and mobile phase B was Acetonitrile (0.1%FA). A Thermo Fisher-TSQ Vantage controlled by X-Caliber was used to perform LC-MRM/MS. Acquisition parameters were: Spray Voltage (+ve) 3500V, Vaporizer temp 100°C 3, Sheath gas flow rate 20 Arb, Auxillary gas flow rate 10Arb, peak width settings 0.70 FWHM, scan time 0.05s, injector settings 0-2mins:waste, 2-16mins:load, 16-20mins:waste. Data was visualized and quantification was performed using X-Caliber software.

Protein –Protein Interaction (PPI) Network Analysis

To identify any reported interaction among the differentially regulated proteins, specifically with S100A8 and S100A9, we performed PPI analysis using 42 differential serum proteins using Network Analyst tool (http://www.networkanalyst.ca/)[4]. String Interactome with confidence score cut-off of 900 was chosen as a PPI database. Further, two types of network analysis was done: 1) zero order network, where only

seed proteins were accessed for interaction among them 2) first order network, where reported possible interactions with any other proteins were also displayed. Importance of the node is decided by 1) degree centrality that is number of connections a node has 2) betweenness centrality that is shortest path going through the node.

Transcript data analysis for S100A8 and S100A9

Transcript data for S100A8 and S100A9 was obtained from various publically available microarray datasets namely: control (TCGA Agilent n=10, TCGA Affymetrix n=10, GSE22866 n=6, REMBRANDT n=28) and in GBM samples (TCGA Agilent n=572, TCGA Affymetrix n=528, GSE22866 n=40, REMBRANDT Grade II n=65, Grade III n=58, GBM n=227). This data was used for mainly three purposes:

1) to represent the differential regulation of transcript levels of S100A8 and S100A9 as compare to control samples by plotting a scatter graph

2) to analyse the significance of transcript levels of S100A8 and S100A9 in prognosis of GBM, the expression data from TCGA with clinical information (Affymetrix platform, n=518) was subjected to survival analysis

3) To perform sample vise correlation analysis with ESTIMATE scores, xCell microenvironment score, cell type specific gene signature and tumor purity score with the expression data of S100A8 and S100A9 (TCGA Agilent (n=318) and Affymetrix platform (n=416), TCGA RNAseq (n=151).

We utilized expression data provided by TCGA, as well as microenvironment scores on the same TCGA samples provided by following three studies:

I. Tumor Purity Score (Nat Biotechnology 2012)[5]

   Tumor purity score is derived from a computational method called ABSOLUTE. This method infers tumor purity and malignant cell ploidy directly from analysis of somatic DNA alterations. As these alterations are present only in tumor cells not in tumor associated normal cells, therefore higher tumor purity score signifies lesser presence of tumor microenvironment.

II. Estimate Score (Nat Communication 2013)[6]

ESTIMATE (Estimation of STromal and Immune cells in MAlignant Tumor tissues using Expression data) is a tool for predicting tumor purity, and the presence of infiltrating stromal/immune cells in tumor tissues using gene expression data. ESTIMATE also utilised algorithm based on single sample GSEA. Thus, higher the ESTIMATE score, more is the presence of tumor microenvironment.

III. xCell Microenvironment Score (Genome Biology 2017)[7]

In xCell method based single sample GSEA is performed to calculate the abundance on scores of 64 immune cell types and a combined xCell microenvironment score. This method utilized the gene signature data including adaptive and innate immune cells, hematopoietic progenitors, epithelial cells, and extracellular matrix cells. Thus, higher the xCell microenvironment score, more is the presence of tumor microenvironment.

All three types of scores have been generated based on specific algorithms details of which are provided in the given references. Tumor purity and Estimate scores were taken from supplementary tables of the references directly. The scores values for only for TCGA GBM samples from these studies were used them for correlation analysis with the expression of genes in the same samples. xCell microenvironment score and and 64 cell type specific score were obtained from xCell version 1.1(http://xcell.ucsf.edu/) for TCGA GBM samples using RNA seq data as input. A cut-off of $r \leq 0.3$ for spearman correlation and pValue=0.05 was used to get significant pairs of gene – cell type correlation.

Real time PCR

Total RNA isolation was done using TRI reagent (Sigma, U.S.A.). Reverse transcription of 2 µg RNA was performed using High capacity cDNA reverse transcription kit (Life technologies, USA). RT-qPCR was done using the ABI PRISM 7900 HT Sequence Detection System (Life technologies, USA). ΔΔCt method.was used to obtain relative expression of the genes of interest normalized using ATP5G and RPL35 as internal control genes. Primer information is provided below:

Gene Name	Forward Primer	Reverse Primer
S100A8	ATTTCCATGCCGTCTACAGG	TGGCTTTCTTCATGGCTTTT
S100A9	GCACCCAGACACCCTGAACCA	TGTGTCCAGGTCCTCCATGATG
RPL35	ACGCCCGAGATGAAACAG	GGGTACAGCATCACTCGG

ELISA for quantification of S100A8 and S100A9

ELISA for control (n=34) and GBM (n=87) serum samples was performed in 96 well formats as per the manufacturer protocol, in duplicates. Two control samples were considered outlier because of their higher values, so 32 control samples were used for further analysis. For each plate separate protein standards in form of dilution series was used. Representative "**standard curve**" obtained by nonlinear regression analysis, as per the manufacturer protocol, performed using Graph pad prism version 5.01 (GraphPad Software, California, USA), are shown in figure below. For all the standard curves, $r^2>0.98$ was achieved.

Each standard curve was used to determine the value of unknowns that is the serum from control and GBM in its corresponding plate. Standard curves were validated to ensure that the curve fitting model used is in accordance with the concentration response of the assay. This was done by plotting **"residual plot".** As can be seen in the representative figure below, delta distance closer to zero indicates, the predicted values of standard by the model used are closer to the actual values.

Next figure shows the **"precision plot"** providing percentage variance of each concentration used for making dilution of standard, between actual value and the value obtained after back calculating the standard's concentration, depicting less than 20 % variation for most the of the points in all the plates, and reaching maximum 35% to 40% at lower concentrations. In addition to this, as the samples were subjected to ELISA in various plates, an "**inter-plate assay comparison**" was performed by comparing the values of each standard point, across plates. Figure below represents the overlap of the values of each standard point across plates, indicating, values of unknown across plates can be compared.

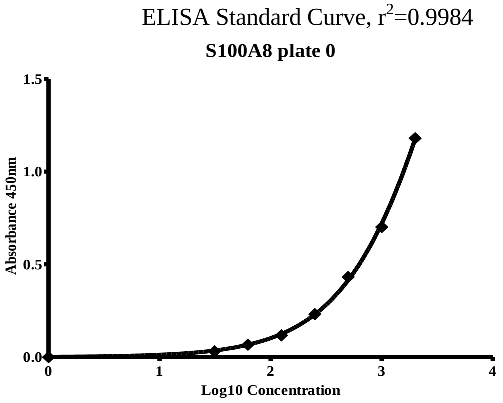

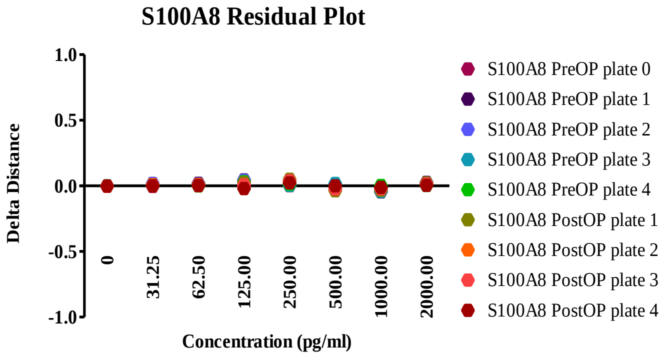

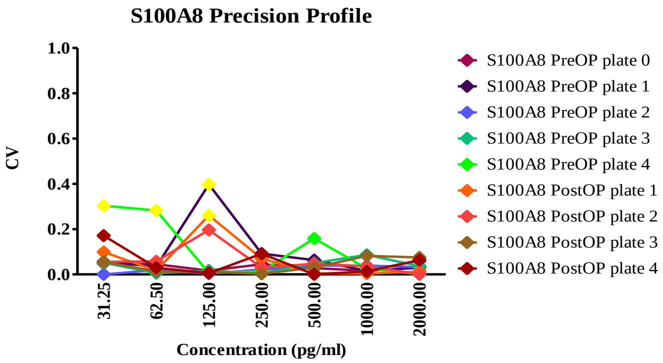

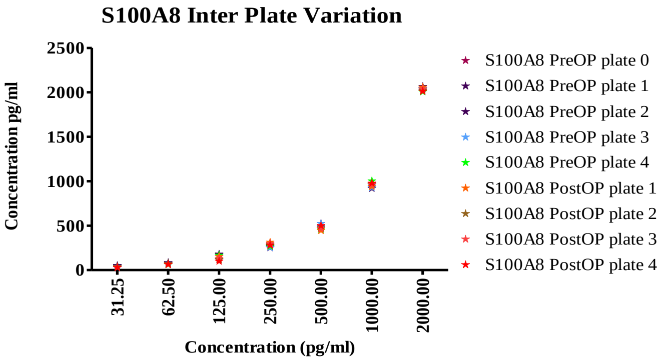

Bacterial purification of S100A8 and S100A9

S100A8 and S100A9 genes were tagged with GST (Glutathione S Transferase) by cloning the full length cDNA in pGEX4T1 vector (GE Healthcare). Briefly, clones obtained were transformed into BL 21 DE3 pLysS expression vector, grown till 0.8 (OD 600) units, induction carried out with 0.5 mM IPTG for six hours, following which cultures were processed by sonication in lysis buffer and the soluble fraction obtained by spinning at 30,000g for 30 minutes. This fraction was incubated with Glutathione Agarose beads (Novagen) and the tagged protein eluted with 10mM reduced Glutathione. Purity was confirmed by Coomassie staining and immunoblot with corresponding antibodies against S100A8, S100A9 (Abcam) and anti-GST antibody (data not shown). The purified protein concentration was quantified in a Nanodrop machine by using molar extinction coefficient values of tagged proteins.

Colony Formation Assay

Colony formation assay was carried out using 6-well culture plate. Briefly, 1000-1500 cells (U251 and T98G) were plated in the 6-well culture plate in serum free conditions along with GST or recombinant proteins- rS100A8 and rS100A9 (0.5 µg/ml), as indicated. After incubation at 37 degree for 12-16 hours, media was changed to complete medium. After 12-15 days, cells were fixed with methanol for 30 minutes, stained with 0.1% crystal violet for 30 minutes, photographed and numbers of colonies were counted.

Migration and Invasion Assay

Migration and Invasion assays were carried out using trans-well Boyden chambers. For measuring the migration potential control inserts were used and for invasion matrigel coated inserts (24-well, Catalogue No. 354578, BD Biosciences) were used. Assay was performed as per manufacture's guide with an optimization for assay time length and number of cells. Briefly, 30000-50000 cells (U373, U138, T98G, U87) were plated in the upper chamber in the serum free conditions along with GST or recombinant proteins- rS100A8 and rS100A9, with vehicle or inhibitor as indicated. Fetal bovine serum (20%) was used in the lower chamber as chemoattractant. After incubation at 37 degree for 12-16 hours, the cells remaining on the upper surface of the membrane were wiped with a wet cotton bud. On the lower surface, migrated and invaded cells were fixed with methanol for 30 minutes, stained with 0.1% crystal violet for 30 minutes, photographed using light microscope and counted. Average was calculated by counting minimum five different fields.

Survival analysis and statistics

The prognostic significance was tested for S100A8 and S100A9 transcript levels in GBM TCGA data (Affymetrix platform, n=518) by performing univariate and multivariate Cox proportional hazard analysis using SPSS software version 19 (IBM Cor., New York, USA). Kaplan–Meier method was used to perform survival analysis at median cut-off of S100A8 and S100A9

transcripts, with a significance calculation done by Log-rank (Mantel–Cox) test by using graph pad prism version 5.01 (GraphPad Software, California, USA).

Receiver Operating Characteristic (ROC) curve analysis was performed using TCGA data for subtypes (Affymetrix, classical (CL), mesenchymal (MES), neural (NE), proneural (PN)), Rambrant transcriptomics data for lower grades (Grade III, GBM) and ELISA data of S100A8 (control n=32 and Grade III=30 and GBM n=87). For subtypes discrimination, analysis was performed by comparing one subtypes to remaining all. Area under curve was obtained and ROC curves were plotted using sensitivities and specificities calculated from all possible cut-off values using SPSS software version 19 (IBM Cor., New York, USA) and graph pad prism version 5.01 (GraphPad Software, California, USA).

Serum levels of S100A8 from patients surviving more than median survival in lab cohort were used for survival analysis using graph pad prism version 5.01 (GraphPad Software, California, USA), significance calculation was done by Log-rank (Mantel–Cox) test. Risk stratification of pre-operative serum samples for patients surviving more than median survival (n=35) was done into three groups low medium and high (20%:20%:60% of total samples respectively). The division of patients for three months post-operative serum levels (n=23) was done on the basis of concentration cut offs of low: medium and high in pre-operative analysis. Graph pad prism version 5.01 (GraphPad Software, California, USA) was used for plotting the graphs.

1 Farrah, T. *et al.* A high-confidence human plasma proteome reference set with estimated concentrations in PeptideAtlas. *Molecular & cellular proteomics : MCP* **10**, M110 006353, doi:10.1074/mcp.M110.006353 (2011).

2 Kusebauch, U. *et al.* Human SRMAtlas: A Resource of Targeted Assays to Quantify the Complete Human Proteome. *Cell* **166**, 766-778, doi:10.1016/j.cell.2016.06.041 (2016).

3 Huang da, W., Sherman, B. T. & Lempicki, R. A. Systematic and integrative analysis of large gene lists using DAVID bioinformatics resources. *Nature protocols* **4**, 44-57, doi:10.1038/nprot.2008.211 (2009).

4 Xia, J., Gill, E. E. & Hancock, R. E. NetworkAnalyst for statistical, visual and network-based meta-analysis of gene expression data. *Nature protocols* **10**, 823-844, doi:10.1038/nprot.2015.052 (2015).

5 Carter, S. L. *et al.* Absolute quantification of somatic DNA alterations in human cancer. *Nature biotechnology* **30**, 413-421, doi:10.1038/nbt.2203 (2012).

6 Yoshihara, K. *et al.* Inferring tumour purity and stromal and immune cell admixture from expression data. *Nature communications* **4**, 2612, doi:10.1038/ncomms3612 (2013).

7 Aran, D., Hu, Z. & Butte, A. J. xCell: digitally portraying the tissue cellular heterogeneity landscape. *Genome biology* **18**, 220, doi:10.1186/s13059-017-1349-1 (2017).

Supplementary figure legends

Supplementary Figure 1:

A, B, C) MARS HU-14 column was used to deplete 14 high abundant proteins. HPLC profile of blank, control pooled sera and GBM pooled sera showing peaks for low abundant protein (LAP) and high abundant protein (HAP).

D) 10 µg of control and GBM pooled sera was loaded on a 10% SDS PAGE gel before (crude serum) and after depletion (LAP and HAP) and silver staining was performed to visualize the removal of HAP. Lane; 1) marker 2) crude control pooled serum 3) control pooled LAP 4) control pooled HAP 5) crude GBM pooled serum 6) GBM pooled LAP 7) GBM pooled HAP.

Supplementary Figure 2:

Theoretical digestion of human proteome was performed and isoelectric point (PI) was calculated for all the peptides, using EXPASY tool.

A) Distribution of theoretically digested peptides across the PI range of 3 to 12 divided into 24 fractions.

B) Pooling of the 24 fractions into 12 fractions on the basis of presence of number of theoretically digested peptides to ensure equal representation in all fractions. After iTRAQ analysis

C) Number of peptides obtained in each fraction

D) Number of proteins obtained in each fraction

E) Average number of peptides and proteins in all fractions showing minimal standard deviation.

Supplementary Figure 3:

A, B) Highlighted selected peptides Peptide I and Peptide II for MRM for S100A8 and Peptide III and Peptide IV for S100A9, in their protein sequence.

C) Confirmation of synthesized peptides, both, unlabelled light peptides (Peptide I, Peptide II Peptide III and Peptide IV) and isotope labelled SIS peptides (Peptide I*, Peptide II*, Peptide III* and Peptide IV*) by MS and MS/MS.

Supplementary Figure 4:

A, B) Calibration curves for S100A8 and S100A9 were performed. Calibration equation and coefficient of regression r^2 is indicated with weighting (w) set as equal.

C) Representative control and GBM MRM profile, for four endogenous peptides (Peptide I, Peptide II for S100A8, Peptide III and Peptide IV for S100A9) and corresponding four SIS peptides (Peptide I*, Peptide II*, for S100A8 and Peptide III* and Peptide IV* for S100A9) used as internal standards.

Supplementary Figure 5:

A) Heat map analysis depicting the correlation between tumor transcripts of 7 high abundant proteins in GBM sera (which correlated significantly with stromal scores and tumor purity score) and xCell cell-type specific signature obtained by xCell method using TCGA GBM RNAseq data. Out of the 64 cell types provided by xCell method only those which gave significant correlation with at least one of the 7 genes were used to plot the heat map. * represents the significant correlation with p value<0.05. Spearman correlation values are colour coded, red for positive correlation, black for no correlation and green for negative correlation.

B, C) Correlation plot between transcript levels of myeloid cell surface antigen CD33 with transcript levels of S100A8 and S100A9. Correlation coefficient and p value, calculated by Spearman's correlation are indicated, dotted lines represent 95% confidence interval.

Supplementary Figure 6:

A, B) Transcript levels of S100A8 and S100A9 in GBM subtypes, classical (CL), mesenchymal (MES), neural (NE), proneural (PN) are shown. ANOVA was performed for significance test and p value less than 0.05 is considered significant with *, **, *** representing p value less than 0.05, 0.01 and 0.001 respectively.

C, D) Transcript levels of S100A8 and S100A9 in TCGA Agilent and Affymetrix datasets, showing higher expression in IDH1 wildtype (IDH1 wt) as compared to IDH1 mutant (IDH1 mut) Unpaired t-test with Welch's correction was performed between IDH1 wt and IDH1 mut GBM samples, p values are indicated, p < 0.05 is represented with *p < 0.01 is represented as ** and p < 0.001 is represented as ***.

E, G, I) Distribution of ESTIMATE score, xCell microenvironment score and tumor purity score, in GBM subtypes, classical (CL), mesenchymal (MES), neural (NE), proneural (PN) are shown. ANOVA was performed for significance test and p value less than 0.05 is considered significant with *, **, *** representing p value less than 0.05, 0.01 and 0.001 respectively.

F, H, J) Distribution of ESTIMATE score, xCell microenvironment score and tumor purity score, monocyte score and Th1-helper cell score in TCGA Agilent datasets, in IDH1 wildtype (IDH1 wt) as compared to IDH1 mutant (IDH1 mut) are shown. Unpaired t-test with Welch's correction was performed between IDH1 wt and IDH1 mut GBM samples, p values are indicated, p < 0.05 is represented with *p < 0.01 is represented as ** and p < 0.001 is represented as ***.

K) Correlation of transcript levels and serum protein levels of S100A8 and S100A9 in various publically available datasets and in our cohort are shown. Correlation coefficient (r) and p values calculated by Spearman's correlation are indicated.

Supplementary Figure 7:

A) ROC curve depicting serum levels of S100A9 do not discriminate between healthy control and GBM, AUC and p value is indicated.

B, C) Kaplan Meier survival analysis using pre-operative serum levels of S00A8 and S100A9 respectively, in our GBM patients cohort (n=87) that does not give prognostic significance. Log-rank (Mantel–Cox) test was applied and the p value is indicated.

D) Kaplan Meier survival analysis using pre-operative serum levels of S100A9, in our GBM patients cohort surviving more than median survival (n=35) that does not give prognostic significance. Log-rank (Mantel–Cox) test was applied and the p value is indicated.

Supplementary Figure 8:

A, B) Role of exogenously added rS100A8 and rS100A9 (recombinant proteins) on glioma cell lines proliferation was measured using colony formation assay. Representative images of duplicate wells after fixing and staining are shown along with the quantitation. p value less than 0.05 is considered significant, ns=non-significant.

C-F) Role of migratory property of exogenously added rS100A8 and rS100A9 (recombinant proteins) on glioma cell lines was measured using trans-well assay. Representative images of T98G, U373, U138, and U87 cells fixed and stained after migration and invasion respectively are shown along with the quantitation. p value less than 0.05 is considered significant with *, **, *** representing p value less than 0.05, 0.01 and 0.001 respectively.

G) Concentration dependent role on migratory and invasive property of exogenously added S100A8 and S100A9 was measured using trans-well assay. Representative images of U138 cells fixed and stained after migration and invasion respectively are shown.

H, I) Quantitation for migration and invasion capability of U138 in presence of three different concentration (GST=40µg/ml, Medium=0.5 µg/ml S100A8 and 0.5µg/ml S100A9, High=20 µg/ml S100A8 and 20ug/ml S100A9) of exogenous S100A8 and S100A9. p value less than 0.05 is considered significant with *, **, *** representing p value less than 0.05, 0.01 and 0.001 respectively.

Supplementary Figure 9:

First order protein-protein interaction network with 40 differential proteins. This network displays all reported interactions of the seed proteins with any other protein in the database.

Importance of the node is decided by 1) degree centrality that is number of connections a node has depicted by size of the node (larger the size more the degree) 2) betweenness centrality that is shortest path going through the node depicted by colour of the node (Red showing highest and blue showing lowest value.

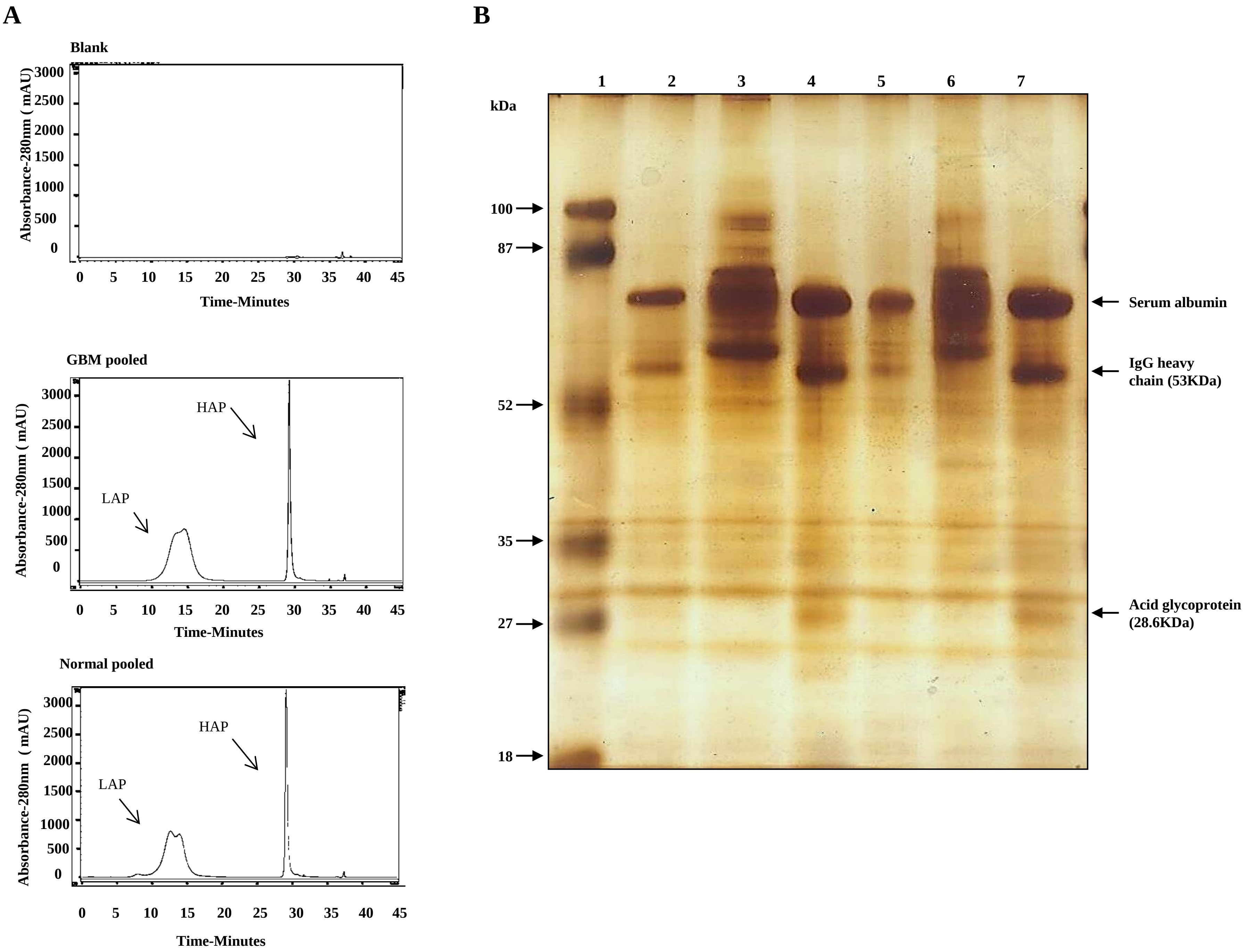
A
Blank
Absorbance-280nm (mAU)
3000
2500
2000
1500
1000
500
0
0 5 10 15 20 25 30 35 40 45
Time-Minutes
GBM pooled
HAP
LAP
Normal pooled
B
1 2 3 4 5 6 7
kDa
100
87
52
35
27
18
Serum albumin
IgG heavy chain (53KDa)
Acid glycoprotein (28.6KDa)

A

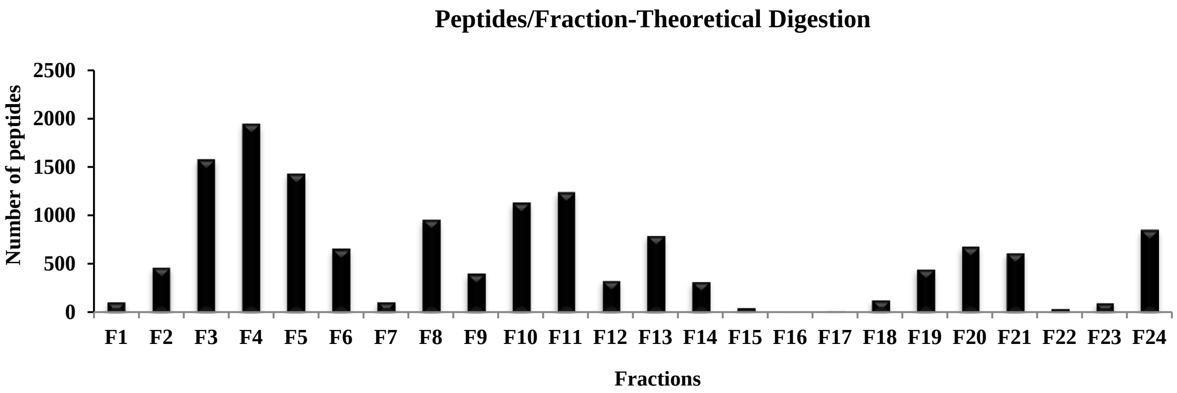

B

iTRAQ Fraction 1	OG-1, OG2
iTRAQ Fraction 2	OG-3
iTRAQ Fraction 3	OG-4
iTRAQ Fraction 4	OG-5
iTRAQ Fraction 5	OG-6, OG-7
iTRAQ Fraction 6	OG-8, OG-9
iTRAQ Fraction 7	OG-10
iTRAQ Fraction 8	OG-11
iTRAQ Fraction 9	OG-12, OG-13, OG-14
iTRAQ Fraction 10	OG-15, OG-16, OG-17, OG-18
iTRAQ Fraction 11	OG-19, OG-20, OG-21
iTRAQ Fraction 12	OG-22, OG-23, OG-24

C

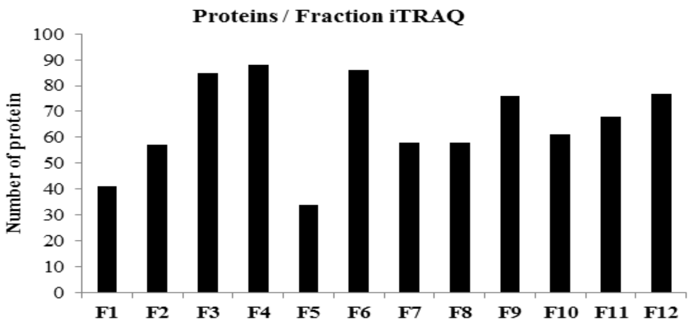

D

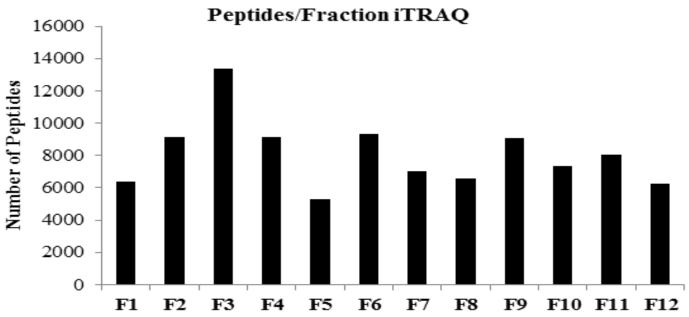

E

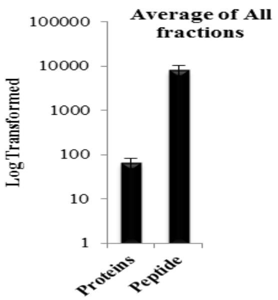

A

>sp|P05109|S10A8HUMAN Protein S100-A8 OS=Homo sapiens GN=S100A8 PE=1 SV=1
MLTELEKALNSIIDVYHKYSLIKGNFHAVYRDDLKKLLETECPQYIRKKGADVWFKELDINTDGAVNFQEFLILVIKMGVAAHKKSHEESHKE

Peptide I-GNFHAVYR , **Peptide II**-MLTELEK

B

>sp|P06702|S10A9HUMAN Protein S100-A9 OS=Homo sapiens GN=S100A9 PE=1 SV=1
MTCKMSQLERNIETIINTFHQYSVKLGHPDTLNQGEFKELVRKDLQNFLKKENKNEKVIEHIMEDLDTNADKQLSFEEFIMLMARLTWASHEKMHEGDEGPGHHHKPGLGEGTP

Peptide III-LGHPDTLNQGEFK, **Peptide IV**-LTWASHEK

C

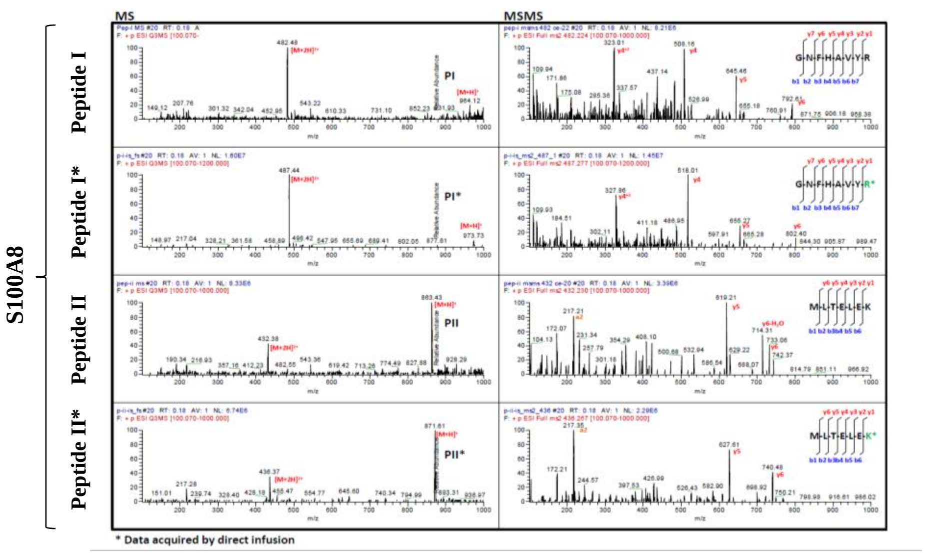

D

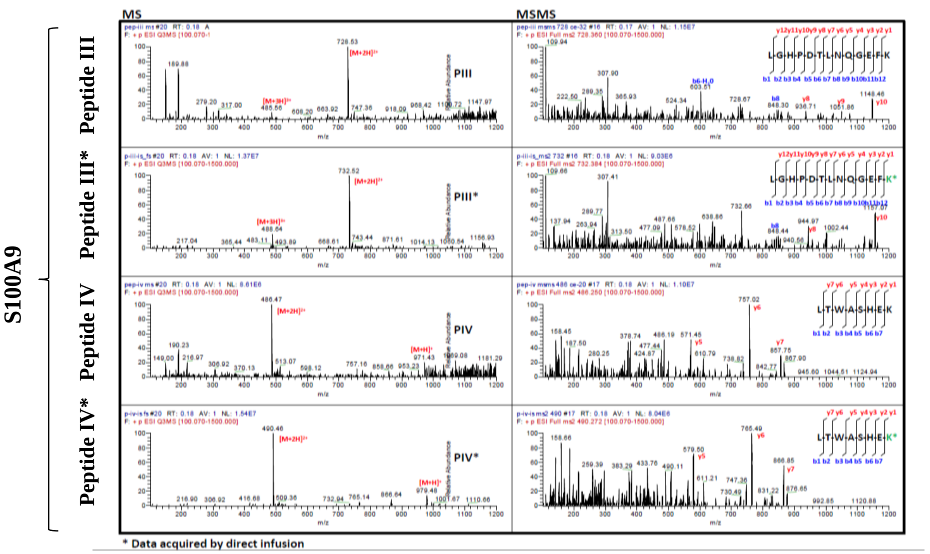

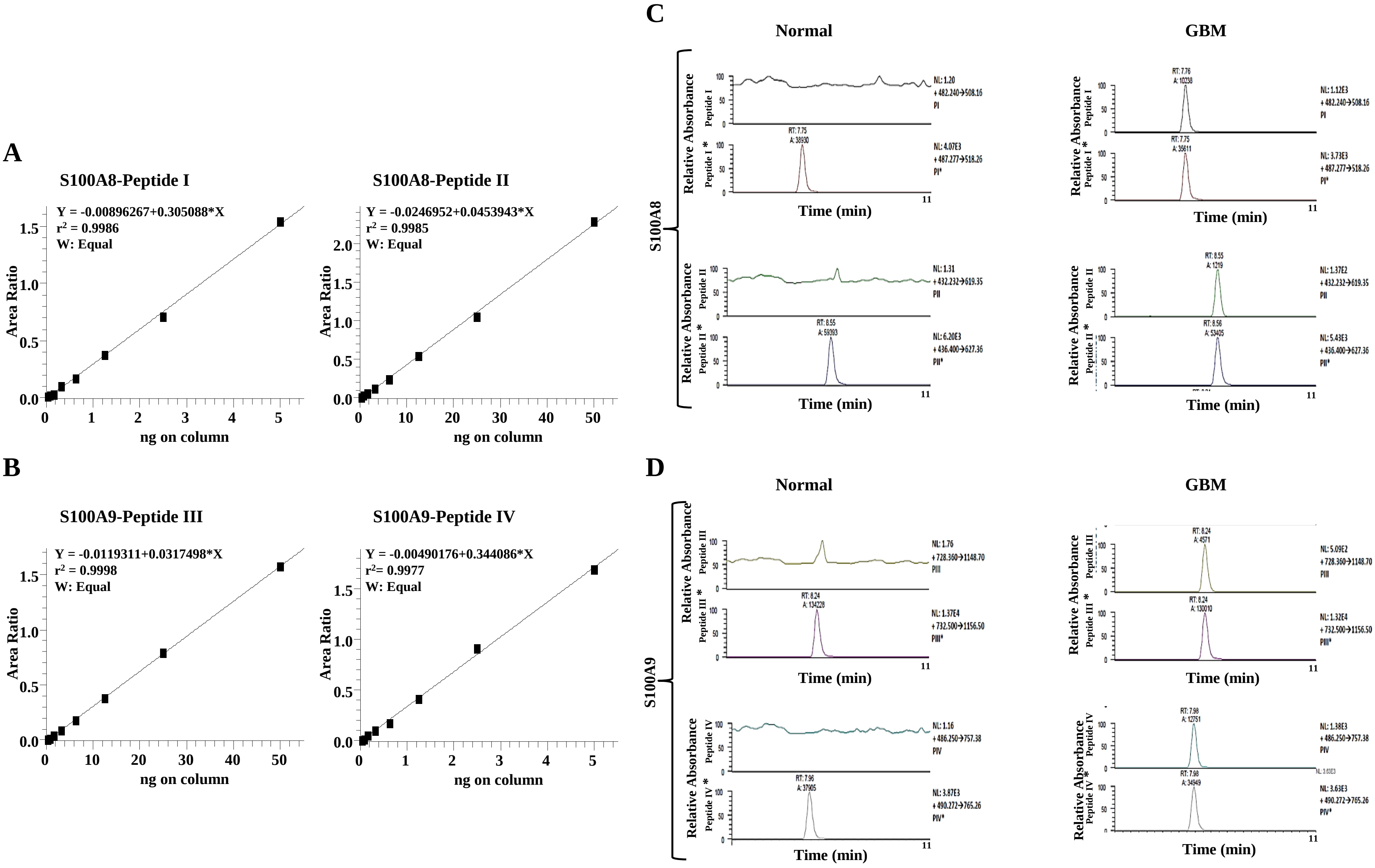
A
S100A8-Peptide I
Y = -0.00896267+0.305088*X
r^2 = 0.9986
W: Equal
Area Ratio
ng on column
S100A8-Peptide II
Y = -0.0246952+0.0453943*X
r^2 = 0.9985
W: Equal
Area Ratio
ng on column
B
S100A9-Peptide III
Y = -0.0119311+0.0317498*X
r^2 = 0.9998
W: Equal
Area Ratio
ng on column
S100A9-Peptide IV
Y = -0.00490176+0.344086*X
r^2= 0.9977
W: Equal
Area Ratio
ng on column
C
Normal
GBM
S100A8
Relative Absorbance
Peptide I
Peptide I *
NL: 1.20
+ 482.240→508.16
PI
RT: 7.75
A: 38930
NL: 4.07E3
+ 487.277→518.26
PI*
RT: 7.76
A: 10238
NL: 1.12E3
+ 482.240→508.16
PI
RT: 7.75
A: 35611
NL: 3.73E3
+ 487.277→518.26
PI*
Time (min)
Peptide II
Peptide II *
NL: 1.31
+ 432.232→619.35
PII
RT: 8.55
A: 59393
NL: 6.20E3
+ 436.400→627.36
PII*
RT: 8.55
A: 1219
NL: 1.37E2
+ 432.232→619.35
PII
RT: 8.56
A: 53405
NL: 5.43E3
+ 436.400→627.36
PII*
D
Normal
GBM
S100A9
Peptide III
Peptide III *
NL: 1.76
+ 728.360→1148.70
PIII
RT: 8.24
A: 134228
NL: 1.37E4
+ 732.500→1156.50
PIII*
RT: 8.24
A: 4571
NL: 5.09E2
+ 728.360→1148.70
PIII
RT: 8.24
A: 130010
NL: 1.32E4
+ 732.500→1156.50
PIII*
Peptide IV
Peptide IV *
NL: 1.16
+ 486.250→757.38
PIV
RT: 7.96
A: 37905
NL: 3.87E3
+ 490.272→765.26
PIV*
RT: 7.98
A: 12751
NL: 1.38E3
+ 486.250→757.38
PIV
RT: 7.98
A: 34949
NL: 3.63E3
+ 490.272→765.26
PIV*

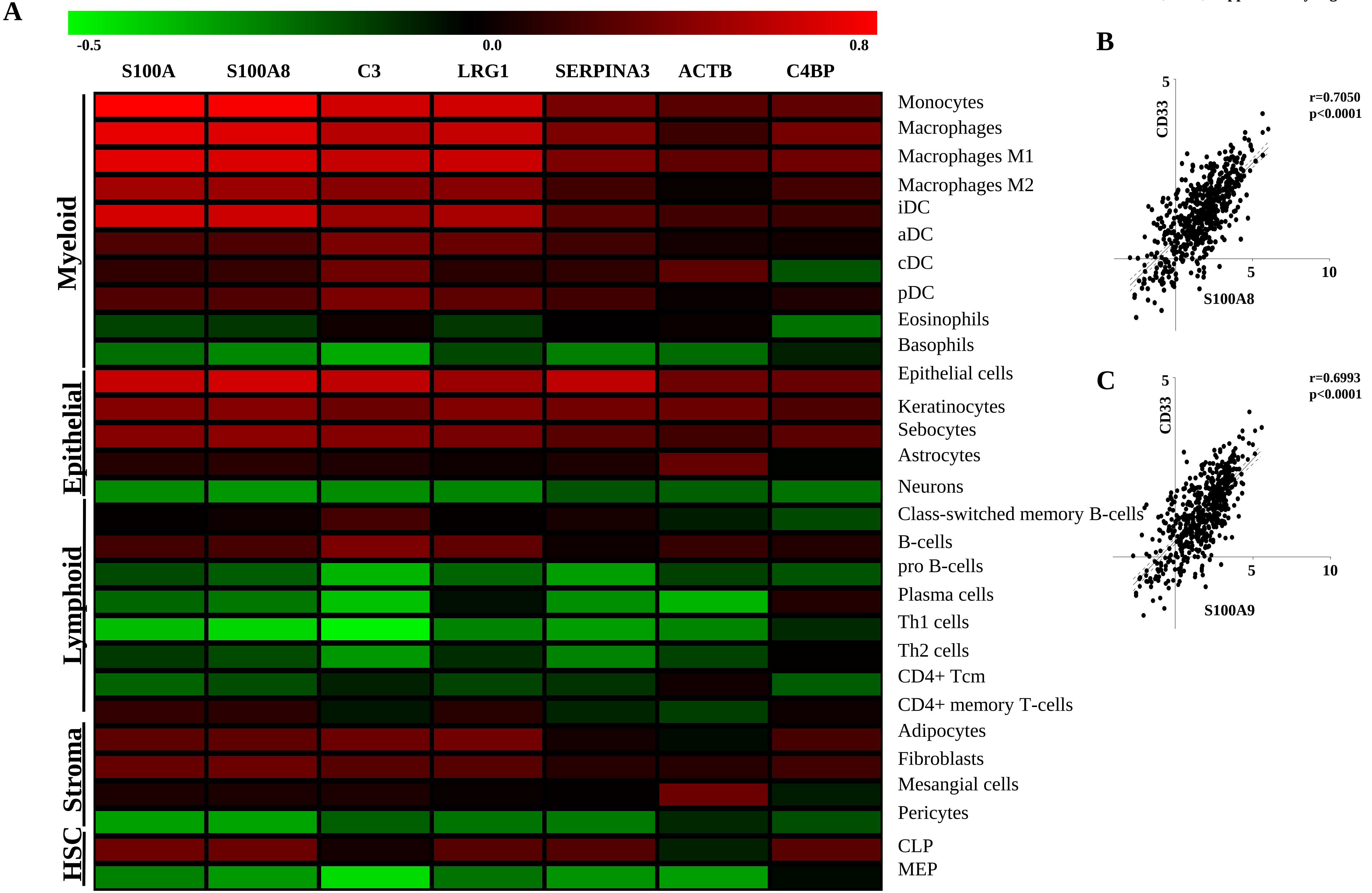
A
-0.5
0.0
0.8
S100A
S100A8
C3
LRG1
SERPINA3
ACTB
C4BP
Myeloid
Epithelial
Lymphoid
Stroma
HSC
Monocytes
Macrophages
Macrophages M1
Macrophages M2
iDC
aDC
cDC
pDC
Eosinophils
Basophils
Epithelial cells
Keratinocytes
Sebocytes
Astrocytes
Neurons
Class-switched memory B-cells
B-cells
pro B-cells
Plasma cells
Th1 cells
Th2 cells
CD4+ Tcm
CD4+ memory T-cells
Adipocytes
Fibroblasts
Mesangial cells
Pericytes
CLP
MEP
B
r=0.7050
p<0.0001
CD33
S100A8
5
5
10
C
r=0.6993
p<0.0001
CD33
S100A9
5
5
10

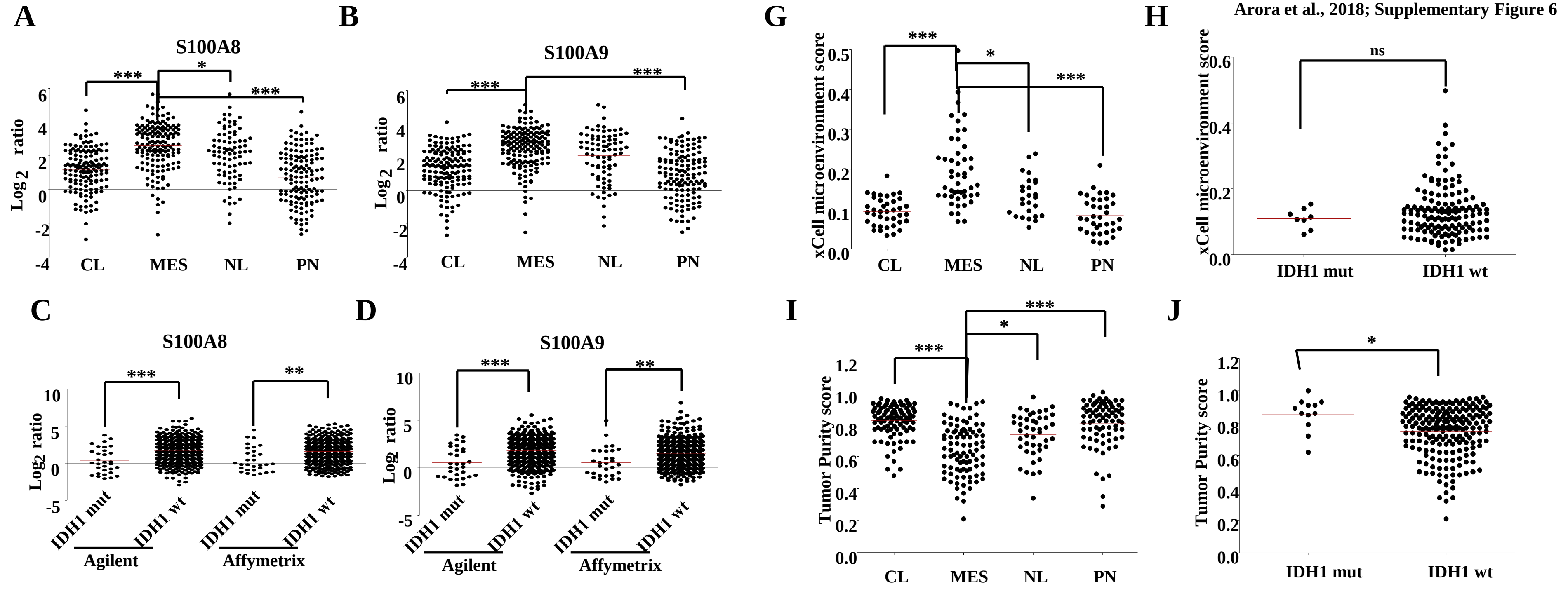

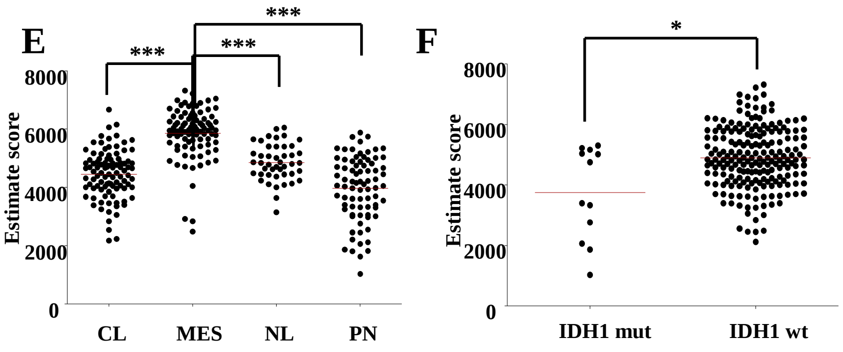

K

Correlation between S100A8 and S100A9 expression						
Sr. No.	Data set	Data type	Samples	p value	Spearman r	95 % CI for Spearman r
1	TCGA Agilent	mRNA microarray	572	< 0.0001	0.954	0.945 to 0.960
2	TCGA Affymetrix	mRNA microarray	528	< 0.0001	0.920	0.906 to 0.933
3	Rembrandt	mRNA microarray	227	< 0.0001	0.886	0.853 to 0.912
4	GSE22866	mRNA microarray	40	< 0.0001	0.855	0.737 to 0.923
5	GSE7696	mRNA microarray	70	< 0.0001	0.899	0.839 to 0.937
6	Our cohort	mRNA RT-q PCR	130	0.8394	< 0.0001	0.777 to 0.884
7	Our cohort	ELISA	87	0.4379	< 0.0001	0.246 to 0.596
8	Our cohort	MRM-PeptideI vs PeptideIII	36	0.8828	< 0.0001	0.776 to 0.940
9	Our cohort	MRM-PeptideI vs PeptideIV	36	0.7533	< 0.0001	0.557 to 0.869
10	Our cohort	MRM- PeptideII vs PeptideIII	36	0.7387	< 0.0001	0.534 to 0.861
11	Our cohort	MRM-PeptideII vs PeptideIV	36	0.5699	0.0003	0.287 to 0.761

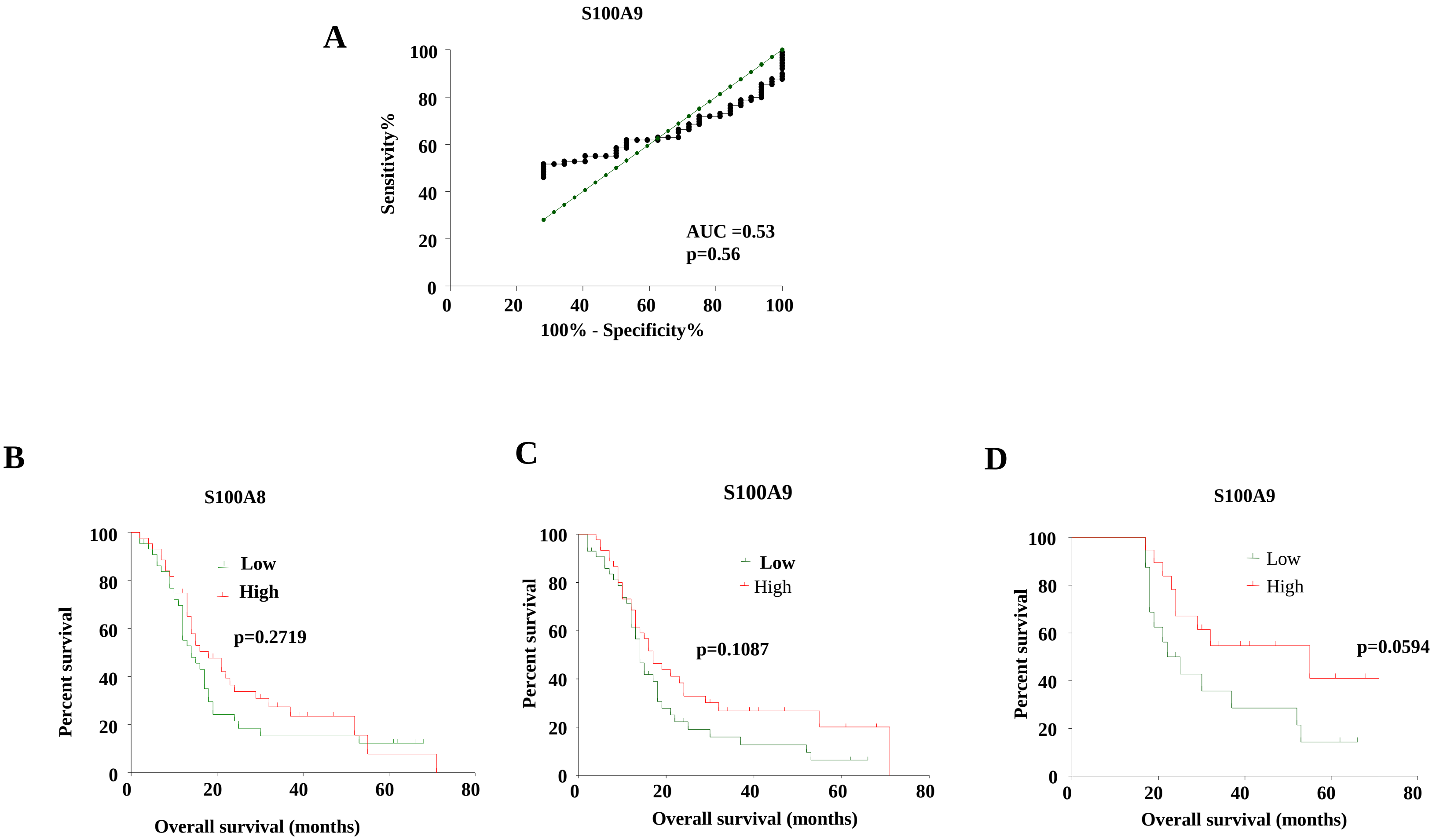
A
S100A9
Sensitivity%
100% - Specificity%
AUC =0.53
p=0.56
B
S100A8
Low
High
p=0.2719
Percent survival
Overall survival (months)
C
S100A9
Low
High
p=0.1087
Percent survival
Overall survival (months)
D
S100A9
Low
High
p=0.0594
Percent survival
Overall survival (months)

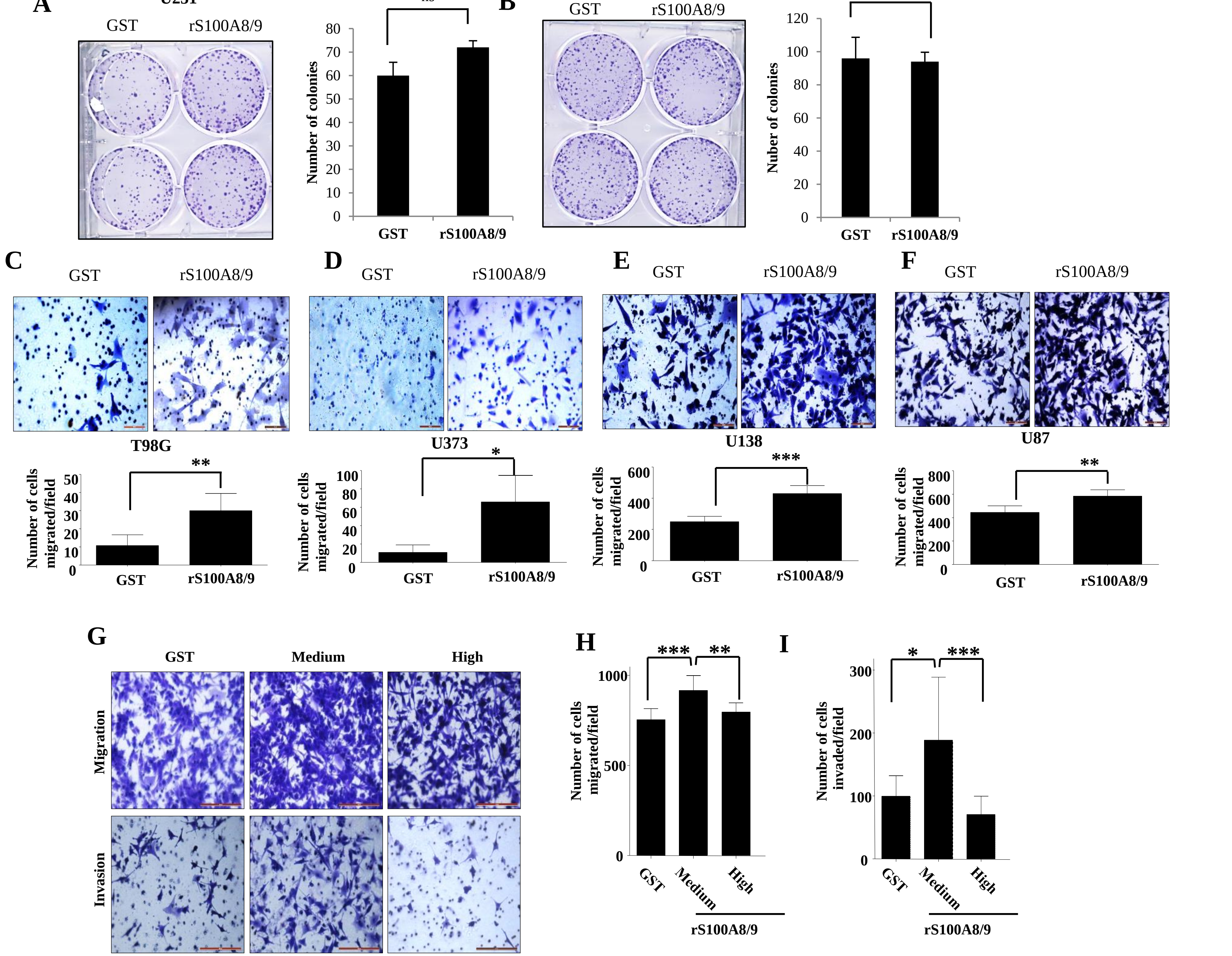
A
U251
GST
rS100A8/9
ns
Number of colonies
80
70
60
50
40
30
20
10
0
GST
rS100A8/9
B
T98G
GST
rS100A8/9
ns
Nuber of colonies
120
100
80
60
40
20
0
GST
rS100A8/9
C
GST
rS100A8/9
T98G
**
Number of cells migrated/field
50
40
30
20
10
0
GST
rS100A8/9
D
GST
rS100A8/9
U373
*
Number of cells migrated/field
100
80
60
40
20
0
GST
rS100A8/9
E
GST
rS100A8/9
U138
***
Number of cells migrated/field
600
400
200
0
GST
rS100A8/9
F
GST
rS100A8/9
U87
**
Number of cells migrated/field
800
600
400
200
0
GST
rS100A8/9
G
GST
Medium
High
Migration
Invasion
H
***
**
Number of cells migrated/field
1000
500
0
GST
Medium
High
rS100A8/9
I
*
***
Number of cells invaded/field
300
200
100
0
GST
Medium
High
rS100A8/9

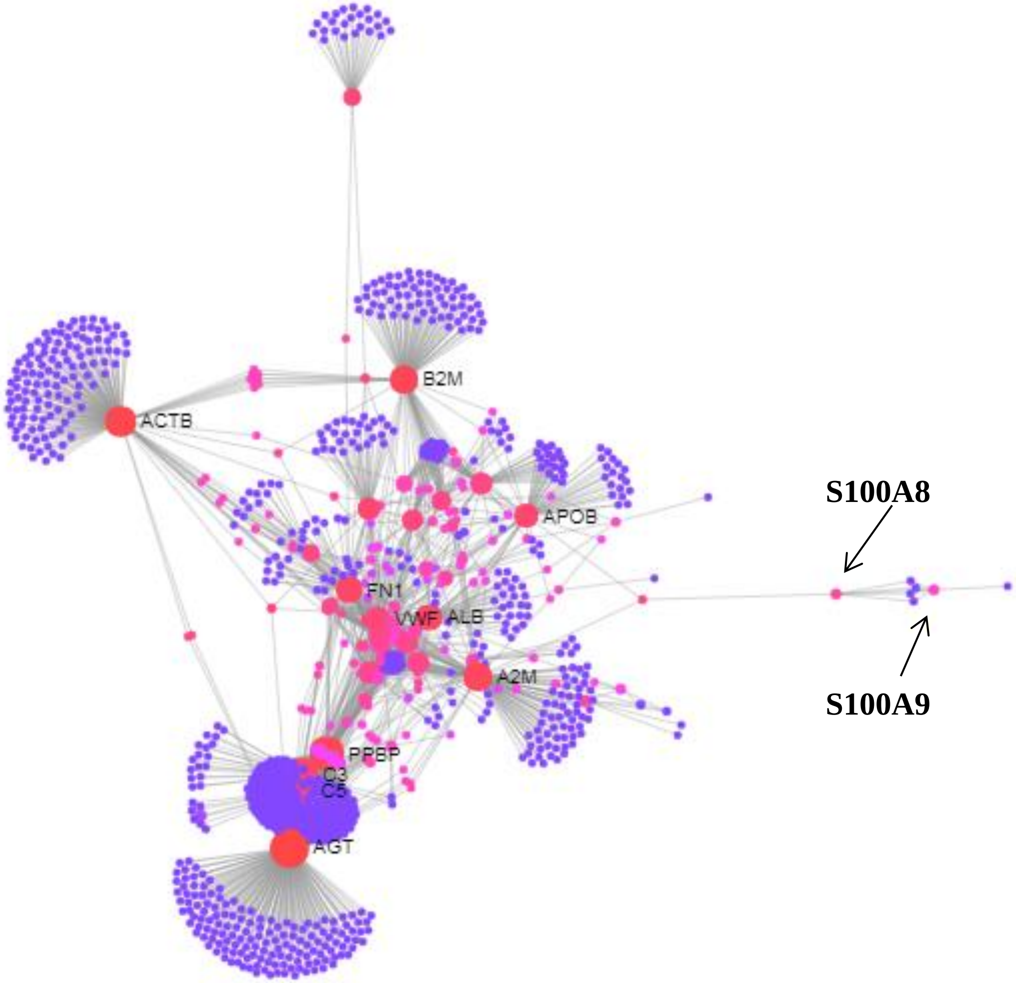
B2M
ACTB
APOB
FN1
VWF
ALB
A2M
PPBP
C3
C5
AGT
S100A8
S100A9
